# Genome-wide association of polycystic ovary syndrome implicates alterations in gonadotropin secretion in European ancestry populations

**DOI:** 10.1038/ncomms8502

**Published:** 2015-08-18

**Authors:** M. Geoffrey Hayes, Margrit Urbanek, David A. Ehrmann, Loren L. Armstrong, Ji Young Lee, Ryan Sisk, Tugce Karaderi, Thomas M. Barber, Mark I. McCarthy, Stephen Franks, Cecilia M. Lindgren, Corrine K. Welt, Evanthia Diamanti-Kandarakis, Dimitrios Panidis, Mark O. Goodarzi, Ricardo Azziz, Yi Zhang, Roland G. James, Michael Olivier, Ahmed H. Kissebah, Reproductive Medicine Network, Ruben Alvero, Huiman X. Barnhart, Valerie Baker, Kurt T. Barnhart, G. Wright Bates, Robert G. Brzyski, Bruce R. Carr, Sandra A. Carson, Peter Casson, Nicholas A. Cataldo, Gregory Christman, Christos Coutifaris, Michael P. Diamond, Esther Eisenberg, Gabriella G. Gosman, Linda C. Giudice, Daniel J. Haisenleder, Hao Huang, Stephen A. Krawetz, Scott Lucidi, Peter G. McGovern, Evan R. Myers, John E. Nestler, Dana Ohl, Nanette Santoro, William D. Schlaff, Peter Snyder, Michael P. Steinkampf, J. C. Trussell, Rebecca Usadi, Qingshang Yan, Heping Zhang, Elisabet Stener-Victorin, Richard S. Legro, Andrea Dunaif

**Affiliations:** 1 Division of Endocrinology, Metabolism, and Molecular Medicine, Department of Medicine, Northwestern University Feinberg School of Medicine, Chicago, Illinois 60611, USA; 2 Center for Genetic Medicine, Northwestern University Feinberg School of Medicine, Chicago, Illinois 60611, USA; 3 Department of Anthropology, Northwestern University, Evanston, Illinois 60208, USA; 4 Section of Endocrinology, Diabetes, and Metabolism, The University of Chicago, Chicago, Illinois 60637, USA; 5 Wellcome Trust Centre for Human Genetics, University of Oxford, Oxford OX3 7BN, UK; 6 Warwick Medical School, University of Warwick, Warwick CV4 7AL, UK; 7 Oxford Centre for Diabetes, Endocrinology and Metabolism, University of Oxford, Oxford OX3 7LE, UK; 8 Oxford NIHR Biomedical Research Centre, Churchill Hospital, Headington OX3 7LE, UK; 9 Institute of Reproductive & Developmental Biology, Hammersmith Hospital, Imperial College London, London W12 0NN, UK; 10 Program in Medical and Population Genetics, Broad Institute of Harvard and MIT, Cambridge, Massachusetts 02142, USA; 11 Division of Endocrinology, Metabolism and Diabetes, University of Utah, Salt Lake City, Utah 84112, USA; 12 Endocrinology and Metabolism, University of Athens Medical School, Athens 115 27, Greece; 13 Division of Endocrinology and Human Reproduction, 2nd Department of Obstetrics and Gynecology, Aristotle University of Thessaloniki 54124, Greece; 14 Division of Endocrinology, Diabetes and Metabolism, Department of Medicine, Cedars-Sinai Medical Center, Los Angeles, California 90048, USA; 15 Departments of Obstetrics and Gynecology and Medicine, Medical College of Georgia, Georgia Regents University, Augusta, Georgia 30912, USA; 16 TOPS Obesity and Metabolic Research Center, Department of Medicine, Medical College of Wisconsin, Milwaukee, Wisconsin 53226, USA; 17 Human and Molecular Genetics Center, Medical College of Wisconsin, Milwaukee, Wisconsin 53226, USA; 18 Department of Genetics, Texas Biomedical Research Institute, San Antonio, Texas 78256, USA; 19 Department of Physiology and Pharmacology, Karolinska Institutet, SE-171 77 Stockholm, Sweden; 20 Department of Obstetrics and Gynecology, Penn State College of Medicine, Hershey, Pennsylvania 17033, USA; 21 Department of Obstetrics and Gynecology, University of Colorado, Aurora, Colorado 80045, USA; 22 Department of Biostatistics & Bioinformatics, Duke University Medical Centre, Durham, North Carolina 27710, USA; 23 Department of Obstetrics and Gynecology, Stanford University Medical Centre, Stanford, California 94304, USA; 24 Department of Obstetrics and Gynecology, University of Pennsylvania School of Medicine, Philadelphia, Pennsylvania 19104, USA; 25 Department of Obstetrics and Gynecology, University of Alabama, Birmingham, Alabama 35249, USA; 26 Department of Obstetrics and Gynecology, University of Texas Health Science Centre, San Antonio, Texas 78229, USA; 27 Department of Obstetrics and Gynecology, University of Texas Southwestern Medical Center, Dallas, Texas 75390, USA; 28 Department of Obstetrics and Gynecology, Baylor College of Medicine, Houston, Texas 77030, USA; 29 Department of Obstetrics and Gynecology, University of Vermont, Burlington, Vermont 05495, USA; 30 Department of Obstetrics and Gynecology, University of Michigan, Ann Arbor, Michigan 48109, USA; 31 Department of Obstetrics and Gynecology, Wayne State University, Detroit, Michigan 48201, USA; 32 Fertility and Infertility Branch, Eunice Kennedy Shriver National Institute of Child Health and Human Development, Rockville, Maryland 20852, USA; 33 Department of Obstetrics Gynecology, and Reproductive Sciences, University of Pittsburgh, Pittsburgh, Pennsylvania 15213, USA; 34 Department of Obstetrics Gynecology, and Reproductive Sciences, University of California at San Francisco, San Francisco, California 94143, USA; 35 Ligand Core Laboratory, University of Virginia Center for Research in Reproduction, Charlottesville, Virginia 22908, USA; 36 Department of Biostatistics, Yale University School of Public Health, New Haven, Connecticut 06520, USA; 37 Department of Obstetrics and Gynecology, Virginia Commonwealth University, Richmond, Virginia 23298, USA; 38 Department of Obstetrics, Gynecology and Women's Health, University of Medicine and Dentistry of New Jersey, Newark, New Jersey 07103, USA; 39 Department of Obstetrics and Gynecology, Duke University Medical Center, Durham, North Carolina 27710, USA; 40 Department of Internal Medicine, Virginia Commonwealth University School of Medicine, Richmond, Virginia 23298, USA; 41 Department of Urology, State University of New York Upstate Medical University, Onondaga, New York 13210, USA; 42 Carolinas Medical Center, Charlotte, North Carolina 28204, USA

## Abstract

Polycystic ovary syndrome (PCOS) is a common, highly heritable complex disorder of unknown aetiology characterized by hyperandrogenism, chronic anovulation and defects in glucose homeostasis. Increased luteinizing hormone relative to follicle-stimulating hormone secretion, insulin resistance and developmental exposure to androgens are hypothesized to play a causal role in PCOS. Here we map common genetic susceptibility loci in European ancestry women for the National Institutes of Health PCOS phenotype, which confers the highest risk for metabolic morbidities, as well as reproductive hormone levels. Three loci reach genome-wide significance in the case–control meta-analysis, two novel loci mapping to chr 8p32.1 and chr 11p14.1, and a chr 9q22.32 locus previously found in Chinese PCOS. The same chr 11p14.1 SNP, rs11031006, in the region of the follicle-stimulating hormone B polypeptide (*FSHB*) gene strongly associates with PCOS diagnosis and luteinizing hormone levels. These findings implicate neuroendocrine changes in disease pathogenesis.

## Abstract (web summary)

Polycystic Ovary Sydrome is a highly heritable, complex reproductive disorder with unknown underlying genetic factors. Here Hayes and Urbanek *et al*. identify three loci in European women strongly associated with neuroendocrine changes and disease susceptibility.

## Graphical abstract

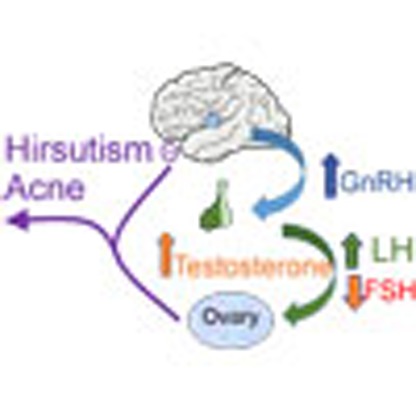

Polycystic ovary sydrome (PCOS) is a common disorder of premenopausal women affecting 7–15% of this population worldwide^1^. It is diagnosed by its reproductive phenotype of hyperandrogenism, ovulatory disturbances and polycystic ovarian changes. PCOS is also frequently associated with insulin resistance, pancreatic β-cell dysfunction and obesity that confer a significantly increased risk for type 2 diabetes (T2D)^1^. The aetiology of PCOS is unknown. Anovulation and hyperandrogenism are a consequence primarily of disordered gonadotropin secretion^1^. Increased LH stimulates ovarian testosterone (T) production while relative FSH deficiency results in arrest of ovarian folliculogenesis^2^. There may also be constitutive increases in ovarian and adrenal androgen secretion^1^. Increased T levels feedback on the hypothalamus contributing to disordered gonadotropin release^3^. Androgen administration during critical developmental windows produces phenocopies of PCOS in animal models suggesting that this mechanism may also be operative in humans^4^. Finally, insulin resistance and the resulting hyperinsulinemia contribute to both hyperandrogenism and ovulatory dysfunction^1^.

In addition, there are abnormalities in the gonadotropin-independent development of preantral follicles in polycystic ovaries (PCO) resulting in an excess of primary follicles compared to normal ovaries^2^. Decreased follicular atresia in PCO may contribute to this finding^2^. However, 20–30% of women with regular menses and no reproductive symptoms have PCO morphology (PCOM)^5^. PCOM is more common in younger women^5^. Even in the absence of hormonal features of the syndrome, women with PCOM have increased sensitivity to exogenous gonadotropin administration^6^.

As the precise cause(s) of PCOS remains elusive, there has been substantial controversy surrounding the most appropriate diagnostic criteria^1^. The first diagnostic criteria, the so-called National Institutes of Health (NIH) criteria, required clinical or biochemical evidence of hyperandrogenism and chronic anovulation with the exclusion of specific conditions that can present with similar features^1^. The Rotterdam criteria^7^ added PCOM as a criterion and required two of three of these features for the diagnosis of PCOS. This modification resulted in multiple phenotypes of affected women, including those with PCOM and hyperandrogenism but no anovulation and those with anovulation and PCOM but no hyperandrogenism. The Androgen Excess Society criteria^8^ modified the Rotterdam criteria to require hyperandrogenism for the diagnosis of PCOS thereby removing the anovulation and PCOM phenotype. There is now general agreement that the NIH criteria identify the phenotype at greatest risk for insulin resistance and its associated metabolic features, whereas the other phenotypes have only minimal metabolic risk^1^,^9^.

Familial aggregation of PCOS and its associated features^10^,^11^ as well as twin studies^12^ have suggested that genetic factors play an important role in disease pathogenesis. Further, two or more phenotypes can be present in the same family suggesting some of these phenotypic differences reflect variable expression and/or penetrance of the same gene^10^. Several susceptibility loci for PCOS have been reproducibly mapped in family-based^13^ and genome-wide association studies^14^,^15^. Previous PCOS genome-wide association studies (GWAS) have been limited to Asian cohorts diagnosed using the Rotterdam criteria^14^,^15^,^16^. PCOS cohorts thus identified have multiple phenotypes of affected women^8^. Therefore, it has not been possible to determine whether the genetic architecture of PCOS varies by phenotype. Further, there have been no genome-wide quantitative trait analyses to assess potential genetic contributions to the hormonal features of the syndrome.

The aim of our study is to identify susceptibility loci for the NIH phenotype of hyperandrogenism and anovulation, which is associated with high risk for insulin resistance and dysglycemia^1^,^9^, and its quantitative reproductive hormone levels^17^. We perform a discovery GWAS (Stage 1) of 984 PCOS cases and 2,964 population controls followed by replication (Stage 2) in 1,799 PCOS cases and 1,231 phenotyped reproductively normal control women. We follow this with a second replication (Stage 3) of the top 24 associations after a meta-analysis of stage 1 and 2 in a cohort of 217 PCOS cases and 1,335 population controls. Three loci reach genome-wide significance in the case–control meta-analysis of all three strata; two novel loci, chr 8p32.1 in the region of *GATA4* and *NEIL2* and chr 11p14.1 in the region of the follicle-stimulating hormone B polypeptide (*FSHB*) gene, and one previously found in Chinese PCOS^15^, chr 9q22.32 in the region of *c9orf3/FANCC*. The same chr 11p14.1 SNP, rs11031006, in the region of the *FSHB* gene also reaches genome-wide significance in the meta-analysis of the quantitative luteinizing hormone (LH) levels. These findings implicate gonadotropins in the pathogenesis of PCOS.

## Results

### Case–control analysis

**Figure 1:**
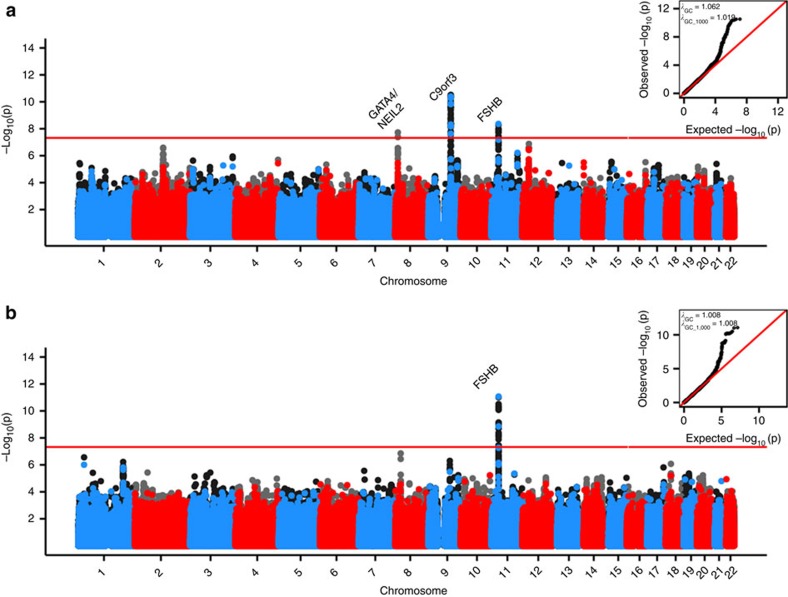
Genome-wide association results for traits with genomewide significant hits in the meta-analysis of GWAS and replication phases. Manhattan plots for **a**. PCOS, **b**. LH levels. Alternating blue and red colours indicate genotyped SNPs, and accompanying black and grey colours indicate imputed variants, on odd and even chromosomes, respectively. The red horizontal red line indicates genomewide significance. QQ plots and *λ*_GC_/ *λ*_GC1000_ are inset in the upper right corner of each plot. For (**a**) PCOS, *P* values are from sample-size weighted two-strata meta-analysis of strata-specific logistic regression *P* values (Stage 1: 984 cases and 2,964 population control women; Stage 2: 1,799 PCOS cases and 1,231 phenotyped reproductively normal control women). For (**b**) LH levels, *P* values are from sample-size weighted two-strata meta-analysis of strata-specific linear regression *P* values (Stage 1: 645 PCOS cases; Stage 2: 399 PCOS cases).

**Figure 2:**
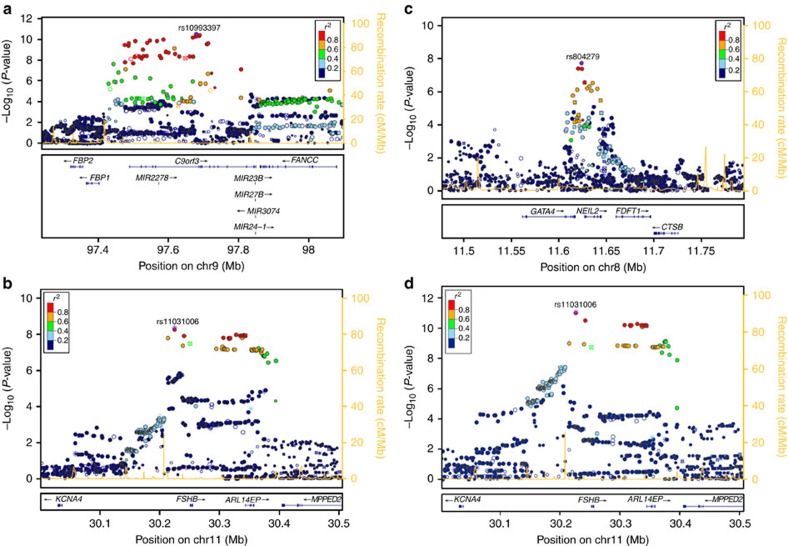
Locuszoom plot of association results, linkage disequilibrium and recombination rates around the genome-wide significant loci. (**a**) Chr. 9 PCOS locus (*c9orf3/FANCC*), (**b**) Chr. 11 PCOS locus (*FSHB/ARL14EP*), (**c**) Chr. 8 PCOS locus (*GATA4/NEIL2*), (**d**) Chr. 11 LH locus (*FSHB/ARL14EP*). In each, the top panel reflects the meta-analysis results of the combined GWAS and replication phases. The LD estimates are colour coded as a heatmap from dark blue (0≥*r*^2^>0.2) to red (0.8≥*r*^2^>1.0). Recombination hotspots are indicated by the yellow lines (recombination rate in cM Mb^-1^ from HapMap (http://hapmap.ncbi.nlm.nih.gov/downloads/recombination/2008-03_rel22_B36/rates/)). The bottom panel shows the genes and their orientation for each region. For (**a**–**c**) PCOS loci, *P* values are from sample-size weighted two-strata meta-analysis of strata-specific logistic regression *P* values (Stage 1: 984 cases and 2,964 population control women; Stage 2: 1,799 PCOS cases and 1,231 phenotyped reproductively normal control women). For (**d**) LH level locus, *P* values are from sample-size weighted two-strata meta-analysis of strata-specific linear regression *P* values (Stage 1: 645 PCOS cases; Stage 2: 399 PCOS cases).

Three loci were associated with PCOS in our cohort (Table 1) at a genome-wide significant threshold after Stage 3: the 8p32.1 *GATA4/NEIL2* locus, the 9q22.32 *c9orf3/FANCC* locus, and 11p14.1 *FSHB/ARL14EP* locus with sample-size weighted three-strata meta-analyses *P*_meta-all_=8.0 × 10^−10^; *P*_meta-all_=4.6 × 10^−13^; and *P*_meta-all_=1.9 × 10^−8^, respectively (Table 2; Figs 1a and 2a–c; Supplementary Data 1, 2).

The 8p32.1 PCOS locus spanned a 6.6 region of extensive linkage disequilibrium (*r*^2^>0.2) including the 3′ end of *GATA4* and all of *NEIL2* into the intergenic region before *FDFT1*. The most strongly associated SNP (rs804279) was intergenic, approached genome-wide significance in the Stage 1 Discovery GWAS (A allele, OR=0.74, 0.66–0.83 95%CI, logistic regression *P*=1.4 × 10^−6^), and was nominally significant in the Stage 2 Metabochip Replication (A allele, OR=0.82, 0.73–0.93 95%CI, logistic regression *P*=2.7 × 10^−3^) for a sample-size weighted two-strata meta-analysis *P*_meta_=1.9 × 10^−8^. The additional UK Replication (Stage 3) was nominally significant (A allele, OR=0.74, 0.58–0.94 95%CI, logistic regression *P*=0.013) for a sample-size weighted three-strata meta-analysis *P*_meta-All_=8.0 × 10^−10^. The proportion of heritability explained by this SNP was 0.79% (ref. 18). After conditioning on the lead SNP in the region, the association was reduced several log orders. This pattern was consistent across all genome-wide significant loci suggesting that there was only one locus within each region of interest discussed below (Supplementary Fig. 1).

The 9q22.32 PCOS locus spanned a ∼331.8 kb extensive linkage disequilibrium (LD) region that includes *c9orf3*, *FANCC* and a number of micro RNA genes. The most strongly associated SNP (rs10993397), in the large fifth intron of *c9orf3*, reached genome-wide significance in the Stage 1 Discovery GWAS (C allele, OR=0.73, 0.66–0.81 95%CI, logistic regression *P*=2.2 × 10^−8^), was also nominally significant in the Stage 2 Metabochip Replication (C allele, OR=0.88, 0.79–0.98 95%CI, logistic regression *P*=2.4 × 10^−4^) for a sample-size weighted two-strata meta-analysis *P*_meta_=3.1 × 10^−11^. This SNP was nominally significant (C allele, OR=0.72, 0.58-0.89 95%CI, logistic regression *P*=3.9 × 10^−3^) in the Stage 3 UK Replication for a sample-size weighted three-strata meta-analysis *P*_meta-All_=4.6 × 10^−13^. The proportion of heritability explained by this SNP was 0.79% (ref. 18). Rs10993397 was 61 kb upstream from the previously identified intronic association in a Chinese population^15^ but still within same the region of strong LD for both European and Chinese ancestry. To test the independence of the two loci, we conditioned the association of rs10993397 with PCOS on the lead Chinese GWAS locus in this region (rs3802457). The association remained genome-wide significant after including the rs3802457 SNP as a covariate in the regression model of PCOS and rs10993397, suggesting that the European and Chinese ancestry specific loci in this region were independent of one another (Supplementary Data 3).

The 11p14.1 PCOS locus spanned a 129.5 kb extensive LD region beginning upstream of *FSHB* and continues through *ARL14EP* (also known as *C11orf46*). The most strongly associated SNP (rs11031006) for both PCOS approached genome-wide significance for PCOS in the Stage 1 Discovery GWAS (G allele, OR=1.41, 1.24–1.62 95%CI, logistic regression *P*=7.8 × 10^−8^) and was nominally significant in Stage 2 Metabochip Replication (G allele, OR=1.18, 1.03–1.37 95%CI, logistic regression *P*=6.3 × 10^−3^) for a sample-size weighted two-strata meta-analysis *P*_meta_=4.3 × 10^−9^. This SNP was not significant in the Stage 3 UK Replication but trended in the same direction as the Stages 1 and 2 (G allele, OR=1.12, 0.83–1.50 95%CI, logistic regression *P*=0.45) for a sample-size weighted three-strata meta-analysis *P*_meta-All_=1.9 × 10^−8^. The proportion of heritability explained by this SNP was 0.80% (ref. 18).

### Quantitative trait analysis

The Stage 1 Discovery GWAS for reproductive hormone levels within the PCOS cases (*N*=645–957 depending on hormone trait) identified three loci reaching genome–wide significance: the 7q33 *DGKI* locus with total T levels, linear regression *P*=1.3 × 10^−8^; the 10q26.11 *CASC2* locus with FSH levels, linear regression *P*=3.2 × 10^−8^; and the 11p14.1 *FSHB/ARL14EP* locus with LH levels, linear regression *P*=1.8 × 10^−9^ (Table 2; Figs 1b and 2d; Supplementary Data 1, 2, Supplementary Figs 2–5). None of these variants showed significant association in the control women (Supplementary Data 4). No genome-wide significant associations with dehydroepiandrosterone sulfate (DHEAS) or sex hormone binding globulin (SHBG) levels were observed (Supplementary Data 1; Supplementary Figs 6 and 7). The same 11p14.1 *FSHB/ARL14EP* SNP, rs11031006, that was most strongly associated with PCOS reached genome-wide significance with LH in the Stage 1 Discovery GWAS (G allele, Beta=3.19, 0.53 s.e., mIU ml^−1^, linear regression *P*=1.8 × 10^−9^) and was nominally significant in the Stage 2 Metabochip Replication (G allele, Beta=2.19, 0.65 s.e., mIU ml^−1^, linear regression *P*=7.7 × 10^−4^) for a sample-size weighted two-strata meta-analysis *P*_meta_=8.5 × 10^−12^. The Stage 3 UK Replication was also nominally significant (G allele, Beta=0.6, 0.12 s.e., mIU ml^−1^, linear regression *P*=1.8 × 10^−6^) for a sample-size weighted three-strata meta-analysis *P*_meta-All_=2.7 × 10^−16^. The proportion of phenotypic variance explained by this SNP was 5.4%. Upon further investigation we determined this high proportion of phenotypic variance explained was largely influenced by several individuals with LH>25 mIU ml^−1^ most likely measured mid-cycle. Trimming the model to only include individuals with LH<25 mIU ml^−1^ the association of rs11031006 with LH levels increased in significance (Stage 1 Discovery GWAS: Beta=3.18, 0.53 s.e., mIU ml^−1^, linear regression *P*=2.0 × 10^−9^; Stage 2 Replication: Beta=1.99, 0.42 s.e., mIU ml^−1^, linear regression *P*=3.3 × 10^−6^; sample-size weighted two-strata meta-analysis *P*_meta_=3.4 × 10^−14^), and the proportion of phenotypic variance explained was reduced to 2.3%.

The 7q33 T locus spans an 8.7 kb extensive LD region within *DGKI*. The most strongly associated SNP (rs706560) reached genome-wide significance in the Stage 1 Discovery GWAS (G allele, Beta =33.29, 5.85 s.e., ng dl^−1^, linear regression *P*=1.3 × 10^−8^). The proportion of phenotypic variance explained by this SNP was 1.9%. The 10q26.11 FSH locus spans a 6 kb extensive LD region that includes *CASC2*. The most strongly associated variant (chr10:119956844:I) reached genome-wide significance in the Stage 1 Discovery GWAS (T allele, Beta=0.27, 0.05 SE, log_10_(mIU ml^−1^), linear regression *P*=3.2 × 10^−8^). The proportion of phenotypic variance explained by this variant was 0.1%. Neither of these SNPs passed imputation quality thresholds in the Stage 2 Metabochip Replication cohort. Further, chr10:119956844:I was not significant in the Stage 3 UK Replication (TCA allele, Beta =0.11, 0.47 s.e., ng dl^−1^, linear regression *P*=0.79), and no longer reached genome-wide significance in the combined meta-analysis (sample-size weighted three-strata meta-analysis *P*_meta-All_=7.3 × 10^−7^). Likewise, rs706560 was not significant in the Stage 3 UK Replication (G allele, Beta =−0.71, 0.47 s.e., ng dl^−1^, linear regression *P*=0.15), and no longer reached genome-wide significance in the combined meta-analysis (sample-size weighted three-strata meta-analysis *P*_meta-All_=6.4 × 10^−6^).

Since BMI itself has a strong genetic component^19^, we explored how our results changed when BMI was removed from the model for the four genome-wide significant loci (Supplementary Data 5). The associations with all four loci (three with PCOS diagnosis, one with LH levels) were only modestly different (less than 1.3 log orders; two increase and two decrease, in linear regression significance) and all remained genome-wide significant. Further the odds ratios for the three PCOS loci changed only 0.01–0.02 in Phase 1 and 0.02–0.07 in Phase 2, which is equivalent to 5.9% change or less. The linear regression beta for the LH locus increased 11 and 22%, respectively, in Stage 1 and 2 when BMI is removed from the model, which suggests that BMI moderately exacerbates the effect of the 11p14.1 LH locus near *FSHB* on LH levels.

We performed an *in silico* look-up of the eQTLs for the three genome-wide significant loci using SCANdb (http://www.scandb.org; Supplementary Data 6). The *c9orf3* locus did not have any eQTLs meeting nominal significance (*P*<1 × 10^−3^) as calculated by the QTDT. The *GATA4/NEIL2* locus had one eQTL meeting nominal significance (*NMUR1*; QTDT *P*=7.0 × 10^−5^), and the *FSHB/ARL14EP* PCOS and LH level locus had four eQTLs meeting nominal significance (*C20orf20*, QTDT *P*=6.0 × 10^−5^, *ARL6IP6*, QTDT *P*=7.0 × 10^−5^; *CCDC69*, QTDT *P*=1.0 × 10^−4^; and *AGPAT2*, QTDT *P*=1.0 × 10^−4^). All of these were detected in European populations (CEU) only. None met a stringent threshold for genome-wide significance.

### Transcriptional analysis

**Figure 3:**
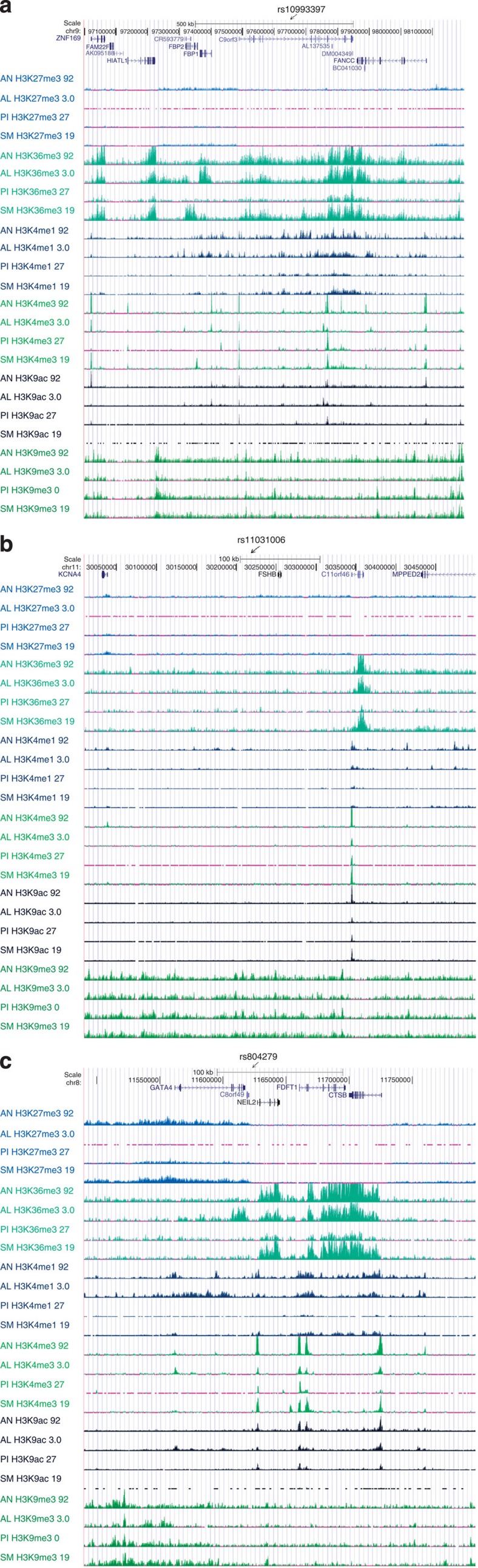
ENCODE histone mark profile for genomewide significant PCOS and LH level GWAS loci (http://www.roadmapepigenomics.org/data). Methylation marks in four PCOS relevant human tissue mapping to: (**a**) Chr. 9 PCOS locus (*c9orf3/FANCC*), (**b**) Chr. 11 PCOS and LH level locus (*FSHB/ARL14EP*) and (**c**) Chr. 8 PCOS locus (*GATA4/NEIL2*). Each is a schematic of the genomic region including diagrams of genes mapping to the region. ChipSeq signal tracks for each tissue (AN, adipose nuclei; AL, adult liver; PI, pancreatic islets; SM, skeletal muscle) for six histone marks (H3K27m3, H3K36m3, H3K4m1, H3K4m3, H3K9ac and H3K9m3) are labelled to the left of the panel.

**Figure 4:**
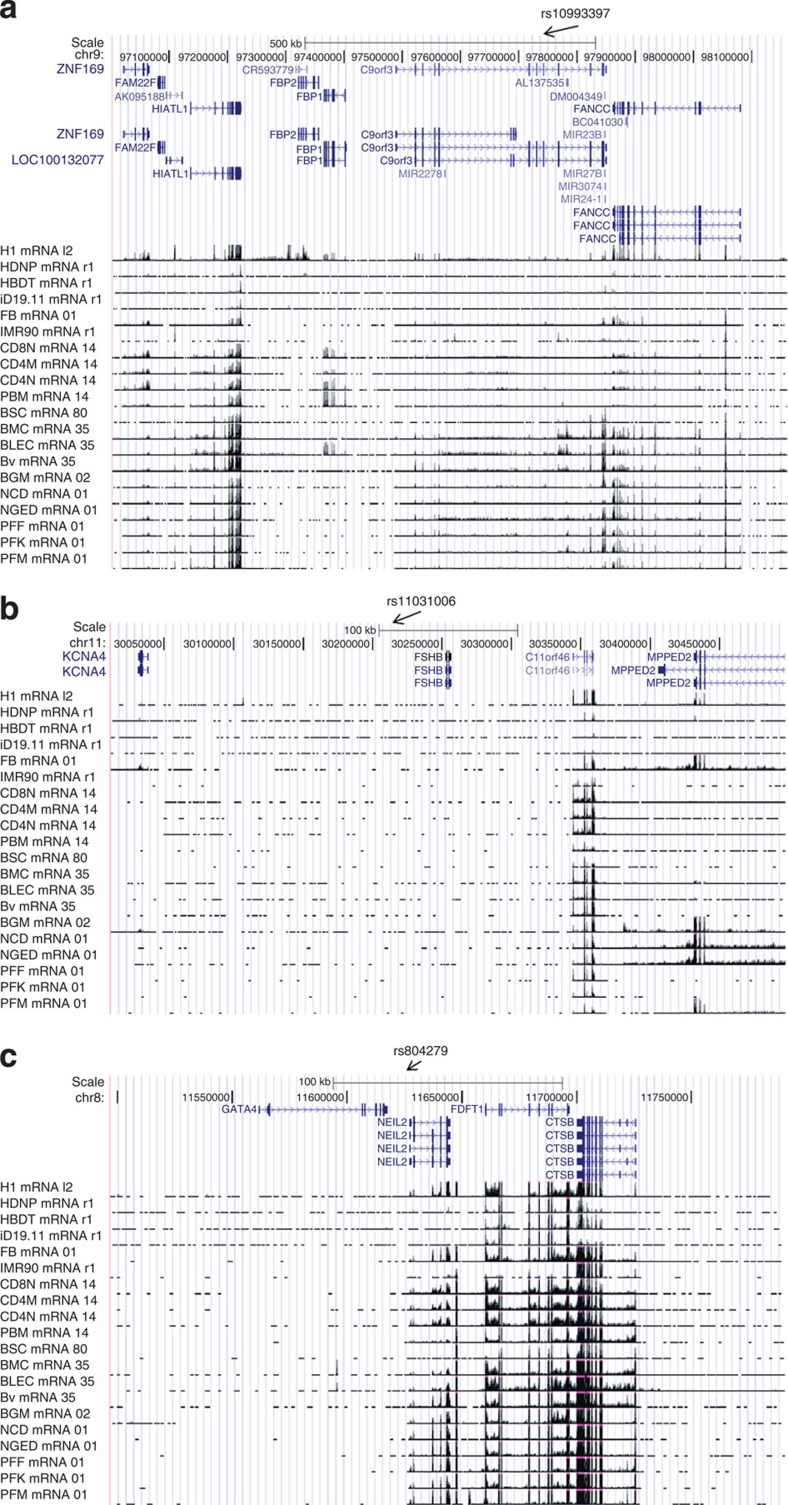
ENCODE mRNA levels for genome-wide significant PCOS and LH level GWAS loci (http://www.roadmapepigenomics.org/data). RNAseq levels in four PCOS relevant human tissue mapping to the (**a**) Chr. 9 PCOS locus (*c9orf3/FANCC*), (**b**) Chr. 11 PCOS and LH level locus (*FSHB/ARL14EP*) and (**c**) Chr. 8 PCOS locus (*GATA4/NEIL2*). Each is a schematic of the genomic region including diagrams of genes mapping to the region. Sources of RNA are labelled to the left of the panel.

There was strong evidence for transcriptional activation as assayed by H3K36m3 and H3K4m3 signals in the chr 9q22.32 *c9orf3/FANCC* PCOS locus (Fig. 3a). Marks indicative of transcriptional activation localized to the 5′ end of *FANCC* and to a ∼100 kb region encompassing a 3′ terminal transcript of *C9orf3* plus a number uncharacterized transcripts in all four ENCODE cell lines (adipose nuclei, adult liver, pancreatic islets and skeletal muscle). Consistent with these observations micro array analysis demonstrated robust *FANCC* expression in the liver, pancreas, and, at low levels, in skeletal muscle, and c9orf3 expression in pancreatic islets and liver (GNF Expression Atlas 1 Human Data on Affy U95 Chips http://genome.ucsc.edu). The RNAseq tracks (http://genome.ucsc.edu; Fig. 4a) demonstrated virtually universal expression of *FANCC*, as well as *HIATl1*, although the latter is outside the bounds of extensive LD from the lead SNP in the region (Fig. 2a).

The chr 8p32.1 PCOS locus was transcriptionally active in adipose nuclei, adult liver, skeletal muscle and pancreatic islets. *NEIL2* and *FDFT1* had evidence for promoter activity via H3K4m3 and H3K9ac binding and evidence of actively transcribed regions via H3K36m3 signals (Fig. 3c). *FDFT1* was expressed at low levels in adipocytes and pancreatic islets and *GATA4* was expressed at low levels in liver and pancreatic islets in microarray analysis (GNF Expression Atlas 1 Human Data on Affy U95 Chips http://genome.ucsc.edu). RNAseq (Fig. 4c) showed absence of expression levels for *GATA4*, inconsistent expression levels of *NEIL2*, and high expression levels of *FDFT1* and *CTSB*, although the latter is outside the bounds of extensive LD from the lead SNP in the region (Fig. 2c).

The most promising functional candidate gene was the *FSHB/ARL14EP* PCOS and LH levels locus mapping to chr 11p14.1. *FSHB* showed no evidence for transcriptional activation in the selected ChipSeq ENCODE (Fig. 3b) or RNAseq cell lines (Fig. 4b), which do not include pituitary tissues. Microarray analysis detected *FSHB* expression predominantly in the pituitary, with low levels detected in adipocytes and the pancreas. Furthermore, *ARL14EP* had evidence for promoter activity (H3K4m3 and H3K9ac) in all four ENCODE cell lines and for actively transcribed genes (H3K36m3) in all cell lines except pancreatic islets. *ARL14EP* was expressed at universally high levels with RNAseq in the cell types shown in Fig. 4b but the microarray analysis detected *ARL14EP* only at low levels in adipocytes (GNF Expression Atlas 1 Human Data on Affy U95 Chips http://genome.ucsc.edu).

Histone mark and mRNA level ENCODE data for the unreplicated 7q33 T (*DGKI*) locus and 10q26.11 FSH (*CASC2*) locus are presented in Supplementary Figs. 8-9.

## Discussion

PCOS is a complex genetic disease characterized by disordered gonadotropin secretion, hyperandrogenism, PCO and insulin resistance^1^. Each of the biochemical features has been hypothesized to play a causal role in the development of PCOS^1^,^4^. There are multiple affected phenotypes depending on the diagnostic criteria applied^1^,^8^. We undertook this study to investigate genetic loci associated with the NIH phenotype of PCOS that confers the highest risk for metabolic morbidities, including T2D and metabolic syndrome^1^,^9^. Three loci reached genome-wide significance in the PCOS case–control meta-analysis, two novel loci, chr 8p32.1 *GATA4/NEIL2* and chr 11p14.1 *FSHB/ARL14EP*, and a chr 9q22.32 *c9orf3/FANCC* locus previously found in Chinese PCOS. The lead SNP at the same novel 11p14.1 *FSHB/ARL14EP* locus, rs11031006, was strongly associated with LH levels in addition to PCOS diagnosis. These findings suggest that genetic variation in FSH plays an important role in the aetiology of PCOS in European ancestry women with the NIH phenotype.

*FSHB* encodes the FSH beta polypeptide^20^, a member of a family of glycoprotein hormones that includes LH, TSH and hCG, and consists of a common alpha subunit and a hormone-specific beta subunit^21^. FSH plays a central role in the regulation of ovarian folliculogenesis, which is disordered in PCOS^2^, in part due to a relative decrease in FSH secretion. LH regulates theca cell T synthesis and increased ovarian androgen production in PCOS is LH-dependent^1^. Adjusting for LH levels in the regression model between rs11031006 and PCOS negated the association (OR=1.18, 1.02–1.37 95%CI, *P*=6.3 × 10^−3^ without LH in the model; OR=1.55, 1.01–2.39 95%CI, *P*=0.66 with LH in the model; Supplementary Data 7), suggesting that the association with *FSHB* is mediated by LH. These findings suggest that variation in *FSHB* contributes to the cardinal gonadotropin secretory changes characteristic of PCOS^22^. Indeed, an *FSHB* promoter polymorphism has been associated with higher circulating LH and lower circulating FSH levels in men^23^, analogous to characteristic changes in gonadotropin levels in PCOS^1^. This polymorphism is also associated with reduced sperm counts^23^. It remains possible that other genes in LD with the 11p14.1 signal, such as *ARL14EP*, which encodes ADP-ribosylation factor-like 14 effector protein that may function to control the movement of MHC class II-containing vesicles^24^, contribute to PCOS pathogenesis. Additional fine-mapping and functional studies will be needed to resolve this issue.

The second novel locus associated with European PCOS was 8p32.1 in the region of *GATA4* and *NEIL2*. *GATA4* encodes a zinc-finger transcription factor that regulates gonadal development and the transcription of steroidogenic genes. Deletion of *GATA4* results in abnormal responses to exogenous gonadotropins and impaired fertility in mice^25^. *GATA4* is expressed in PCOS ovarian follicles but this expression does not differ from control follicles^26^. However, the expression of another member of this family, *GATA6*, is increased in PCOS theca cells^27^. *NEIL2* encodes nei endonuclease VIII-like 2, a member of a class of DNA glycosylases involved in the repair of DNA damage^28^. The 8p32.1 locus also encompasses the promoter region of *FDFT1* that encodes farnesyl-diphosphate farnesyl transferase, the first specific enzyme in the cholesterol biosynthesis pathway and thus also critical for testosterone biosynthesis. *FDFT1* is associated with nonalcoholic fatty liver disease^29^.

We also replicated the 9q22.32 *c9orf3/FANCC* PCOS locus previously reported in Han Chinese^15^, although the Chinese and European loci were independent of one another (Supplementary Data 3). Multi-ethnic fine-mapping will be required to tease this apart further. One gene in this region, *c9orf3*, encodes a member of the M1 zinc aminopeptidase family, aminopeptidase O (AP-O)^30^. AP-O is expressed in the pancreas, placenta, liver, testis and heart where it is postulated to play a role in proteolytic processing of biologically active peptides, for example, the cleavage of angiotensin III to generate angiotensin IV^30^. *FANCC* encodes the protein for Fanconi anaemia complementation group C protein (FANCC) that functions in complexes to repair DNA cross-linking damage, and also suppresses apoptosis^31^.

Further, we nominally replicated (*P*<0.05), in the same direction, seven of the eleven previously identified Chinese PCOS SNPs (Table 3), including gonadotropin receptor genes *LH/CGR* and *FSHR*, as well as *THADA*, *DENND1A*, *YAP1*, and *RAB5B/SUOX*^14^,^15^, exceeding the number of associations we would expect to replicate by chance alone. We did not replicate the previously identified 8q24.2 Korean PCOS locus (Table 3)^16^. However, this GWAS in Korean women with the Rotterdam PCOS phenotypes failed to replicate any of the European or Chinese PCOS loci at a significance level of 10^−5^ or higher^16^. The failure to replicate these loci could be due to a lack of power in the Korean study or to differences in the phenotypes studied, although both the Chinese and Korean GWAS used the Rotterdam diagnostic criteria.

Two quantitative hormone level loci, in addition to the 11p14.1 *FSHB/ARL14EP* LH locus, were genome-wide significant in the Stage 1 Discovery GWAS of quantitative hormone levels: the7q33 T locus, in the region of *DGKI*, and the 10q26.11 FSH locus, in the region of *CASC2*. They failed quality control in the Stage 2 Metabochip Replication cohort and did not demonstrate significant associations in the Stage 3 UK Replication cohort. After inclusion of the Stage 2 and 3 replication data, the meta-analysis across all three strata no longer reached genome-wide significance for these two loci. Further, the locuszoom plot (Supplementary Fig. 5) for the 10q26.11 FSH locus (*CASC2*) indicates only a single variant in the region was strongly associated (*P*<10^−4^), potentially indicating poor imputation of this indel, although the imputation *r*^2^>0.9. Therefore, these findings should be considered tentative, and will require further testing in additional cohorts. *DGKI*, diacylglycerol kinase iota, is a member of the type IV diacylglycerol kinase family^32^. *CASC2*, cancer susceptibility candidate 2, has been associated with endometrial cancer^33^.

A GWAS of T and sex hormone binding globulin (SHBG) levels in men^34^ found an association between variants in the *SHBG* gene and these quantitative hormone levels. We replicated the findings for rs12150660 (Supplementary Table 8) with both T and SHBG levels, but not at genome-wide significant thresholds. We could not attempt to replicate the associations with rs6258 as the genotype data for that SNP did not pass quality control thresholds in our study. As the mean and range of T levels in both PCOS and control women were much smaller (approximately one-tenth that in men), this may have limited the ability to detect associations identified in men^34^.

Increased BMI is commonly associated with PCOS, particularly in US cohorts of European ancestry^1^. Accordingly, we adjusted for BMI in our model to reduce the chance of identifying BMI-related rather than PCOS-related loci. However, removing BMI from the model had minimal impact on our results. All four genome-wide significant loci (three PCOS diagnosis associated, one LH levels associated) were genome-wide significant with or without the inclusion of BMI as a covariate. Removing the BMI covariate led to evenly split number of increases and decreases in significance, and the effect sizes changed <6%. We also did not find any evidence for genome-wide significant associations with PCOS of variants in known obesity or type 2 diabetes genes. Collectively, these findings suggest that while BMI is associated with PCOS, genes involved in BMI have little impact on the genetics of PCOS.

The heritability of PCOS is ∼70% in monozygotic twin studies^12^. However, the portion of heritability accounted for by the three PCOS loci were small (<1% each) as has been observed with other common complex diseases and traits^35^. Although we now count fourteen replicated genome-wide significant PCOS loci across Chinese^14^,^15^ and European^36^,^37^,^38^ ancestry populations including those identified in this study, each has relatively small effect sizes that when taken collectively only account for a small portion of the heritability of PCOS as estimated from twin studies. Investigations of the genome-wide genetic architecture^39^ of PCOS should increase this, but other variables (for example, structural variants, epigenetic factors, epistasis and so on) likely account for the remaining heritability.

We assessed the transcriptional potential of the three PCOS GWAS loci using three independent methods. Each of the three methods capture slightly different data on transcription potential and have different sensitivities to detect transcription^40^,^41^. Data from the two sequencing-based data methods (ChIP-Seq and RNASeq) are more congruent with each other than either are with data from the expression arrays (GNF Expression Atlas 1 Human Data on Affy U95 Chips http://genome.ucsc.edu). Further, the results for any given tissue or cell line are derived from a single individual and thus do not take into account inter individual variation in gene expression (http://genome.ucsc.edu). With these caveats in mind, each of the genomic regions identified in our GWAS was shown to be transcriptionally active in at least one tissue that is affected in PCOS. These findings are consistent with these genomic regions harbouring PCOS susceptibility genes, although clearly further studies in tissues relevant to the PCOS reproductive phenotype are needed.

Our study was limited to women with the NIH phenotype of PCOS that is defined by endocrine features rather than PCOM. We focused on this phenotype because it is the one associated with highest risk for insulin resistance and other metabolic disorders^1^,^9^. Further, ∼90% of women with NIH phenotype PCOS have PCOM^1^,^42^. PCOM is also a common, age-related finding in otherwise reproductively normal women^5^,^42^. Nevertheless, even in the absence of hormonal abnormalities, PCOM appear to have intrinsic abnormalities in folliculogenesis^2^ and gonadotropin responsiveness^6^. PCOS cohorts diagnosed by Rotterdam criteria are enriched for the phenotypes with PCOM^1^ and with less severe endocrine abnormalities^1^,^8^,^9^,^43^. Accordingly, the inclusion of these additional phenotypes may have contributed to the differing results between the present and previous GWAS^15^,^16^. In addition, the fact that we did not exclude PCOM in our control subjects, as was done in the previous Asian PCOS GWAS^15^,^16^, may have limited our power to detect genetic variation associated with PCOM.

In conclusion, replicated GWAS in Chinese^14^,^15^ and European cohorts^36^,^37^,^38^ implicate genes modulating gonadotropin action, *LHCGR* and *FSHR*, and secretion, *FSHB*. These findings suggest that gonadotropins play an aetiologic role in PCOS pathogenesis, analogous to the insights into causative biologic pathways provided by T2D GWAS implicating β-cell genes and obesity GWAS implicating neuroendocrine genes modulating food intake^44^. Further, these PCOS GWAS studies are complementary since the Chinese cohort contained additional Rotterdam phenotypes, whereas our cohort contained only the NIH phenotype with hyperandrogenism and chronic anovulation. The Chinese GWAS findings of associations with the genes encoding gonadotropin receptors, *LHCGR* and *FSHR*, suggest that ovarian gonadotropin action plays a role in all of the Rotterdam phenotypes, while our findings of associations of *FSHB* with PCOS and LH levels suggest that gonadotropin secretion drives the accompanying hormonal derangements of hyperandrogenism and anovulation. Further genetic analyses stratified by Rotterdam phenotype are warranted. In addition, there was a common 9q22.32 PCOS locus in the Chinese and European cohorts, suggesting shared susceptibility variants in these ethnically diverse populations. As human populations ancestral to Chinese and Europeans diverged from Africans ∼60,000 years ago^45^,^46^, the 9q22.32 region may harbour an evolutionarily conserved genetic susceptibility factor for PCOS^47^. Studies in African ancestry PCOS populations should provide further insight into the evolutionary history of this disorder.

## Methods

### Subjects

Nine-hundred eighty-four PCOS cases and 2,964 population controls (Stage 1), followed by replication (Stage 2) in 1,799 PCOS cases and 1,231 phenotyped reproductively normal control women were studied. An additional replication (Stage 3) of the top variant from each region with *P*<5 × 10^−6^ (*N*=24) was performed in 217 PCOS cases and 1,335 1958 British Birth Cohort (that is, controls) samples. The study was approved by theInstitutional Review Board of Northwestern University Feinberg School of Medicine as well as by the Institutional Review Boards of the study investigators' institutions (Institutional Review Boards of Brigham and Women's Hospital, Carolinas Medical Centre, Cedars-Sinai Medical Centre, Pennsylvania State Milton S. Hershey Medical Centre, Magee-Womens Hospital, Medical College of Wisconsin, Massachusetts General Hospital, University of Pennsylvania, Stanford University, University of Alabama at Birmingham, University of Chicago, University of Colorado at Denver, University of Michigan, University of Medicine and Dentistry of New Jersey, University of Texas Health Science Centre, University of Texas Health Science Centre at San Antonio, University of Vermont, Virginia Commonwealth University, Wayne State University; the Research Ethics Committee of Athens University Medical School; and the North Thames Multicenter Research Ethics Committee). All subjects were Caucasians of European ancestry and gave written informed consent before study. Subjects providing samples for the NUgene DNA biorepository consented to the use of their deidentified clinical data and DNA samples by third party investigators^48^.

PCOS subjects were ages 13–45 years (Table 1). Subjects in the discovery cohort (Supplementary Data 9) were recruited by the authors (A.D., R.S.L., D.A.E.) and the Reproductive Medicine Network (RMN) for the Pregnancy in Polycystic Ovary Syndrome (PPCOS)I study^49^. PCOS subjects (Supplementary Data 10) were recruited by the same authors, the RMN PPCOSII study^50^, as well as by collaborations with PCOS research centres across the United States (C.K.W., M.O.G., R.A.) and in Greece (E.D.K., D.P.) for Stage 2 Replication and in the United Kingdom (T.B., M.M., S.F.) for Stage 3 Replication. PCOS cases fulfilled NIH criteria for PCOS^1^. Cases fulfilling these criteria also meet the Rotterdam and Androgen Excess Society criteria for PCOS^1^. The cases had hyperandrogenism (clinical and/or biochemical) and chronic anovulation (eight or fewer menses per year) with exclusion of other hyperandrogenic disorders in the differential diagnosis, such as non-classical adrenal 21-hydroxylase deficiency^1^. Ovarian morphology was not a criterion for the diagnosis as it is a non-specific finding that does not correlate with the endocrine phenotype^5^,^42^.

Control DNA samples for the discovery cohort came from women self-identified as ‘white,' age 18-97 years collected by the NUgene DNA bank^48^. The samples were selected from women with data on body weight, height and age. Control women in the Stage 2 replication cohort were reproductively normal women, ages 15–45 years, with regular menses and no hirsutism who were enroled by the following authors (A.D., R.S.L., C.K.W., E.D-K., D.P., M.O.G., R.A.) as control subjects for their PCOS studies; the control women had T levels within the normal range for the assay used by each author. Questionnaire-based reproductive histories, DNA and EDTA-plasma samples were obtained from 18- to 45-year-old women who were enroled in the Metabolic Risk Complications of Obesity Genes project^51^. Women selected had regular menses and were not receiving oral contraceptive pills. They had normal total T levels determined using liquid chromatography-tandem mass spectrometry (LC-MS/MS)^52^. The details of recruitment and phenotyping of the discovery and replication cohort PCOS cases and the replication cohort control women have been reported previously (see references in Supplementary Data 11). Control women in the UK replication cohort (Stage 3) were from the 1958 British Birth Cohort (58BBC), and all were of the age 44-45 years at the time of DNA sample and phenotypic data collection (BMI and age).

### Hormone level assays

The hormone levels used for subject phenotyping and in the quantitative trait analyses were measured as outlined in Supplementary Data 11 All analyses are adjusted for the study site at which the assay was performed, a central laboratory was used by the RMN and was, therefore, considered as one site. Further, some sites changed assay methodology so the analyses were also adjusted for the assay methodology. The normal range for each assay was established in the following number of premenopausal, reproductively normal, non-hirsute control women with regular menses: 98 Cedars Sinai Medical Centre^53^; 100 University of Alabama at Birmingham^54^; 108 University of Athens^55^; 39 University of Chicago^56^, and 75 United Kingdom^11^.

The normal ranges for the PCOS Family Study were established as reported in 43 premenopausal reproductively normal women with 27–35 day menstrual cycles and no hirsutism at Penn State Milton S. Hershey Medical Centre^10^. Since 2006, the T assay for the PCOS Family Study has been performed at the University of Virginia Centre for Research in Reproduction Ligand Assay and Analysis Core using the same assay method (Supplementary Data 11). The normal ranges were validated in this assay and have been reassessed in an additional cohort of 209 premenopausal reproductively normal women with 27–35 day regular menses and no clinical symptoms of androgen excess to ensure that the range has not changed. The normal range for LC-MS/MS T assays used to select control women enroled in the Metabolic Risk Complications of Obesity Genes project^51^ was also determined in this additional cohort of 209 control women. The assays for PPCOSI and PPCOSII were performed at the University of Virginia Centre for Research in Reproduction Ligand Assay and Analysis Core. Accordingly, the same normal range was used at these study sites as was used for the PCOS Family Study T assays performed in this core.

### Stage 1 Discovery GWAS

PCOS cases and NUgene control DNA samples included in the GWAS phase were genotyped using the Illumina Omni Express (HumanOmniExpress-12v1_C) at the Centre for Inherited Disease Research (CIDR), Johns Hopkins University, Baltimore, MD.

### GWAS Quality control (QC)

Genotypic data that passed initial quality control at the genotyping centre were released to the PCOS study team who performed quality control procedures of the data following recommendations from the GENEVA^57^ and eMERGE^58^,^59^ consortia, in which we were involved. Specifically, we removed poorly performing samples or SNPs based on misspecified sex, chromosomal anomalies, unintended sample duplicates, sample relatedness, low call rate, high number of Mendelian errors, departures from Hardy–Weinberg equilibrium, duplicate discordance, sex differences in heterozygosity and low minor allele frequencies as detailed in Supplementary Data 12 and 13.

### Ancestry

To investigate population structure, we used principal components analysis (PCA) in EIGENSTRAT (http://genepath.med.harvard.edu/~reich/Software.htm), essentially as described by Price *et al*.^60^ We used PCA for two purposes: to identify population group outliers and to include as covariates in the statistical model used for association testing to account for population structure. Initially, we analysed all unduplicated PCOS GWAS study samples separately, along with HapMap (Phase 3 CEU, CHB, JPT and YRI) to detect of population outliers among the study. We started with a set of 703,171 autosomal SNPs with missing call rate <1%. From this pool of overlapping SNPs, we selected a subset through two rounds of LD pruning using the pair-wise genotypic correlation method in PLINK^61^ (http://pngu.mgh.harvard.edu/~purcell/plink/). In the first round, we removed SNPs in short-range LD using a window size of 50 SNPs with a five SNP offset and an *r*^2^ threshold of 0.8. The second round of pruning removed SNPs in long-range LD using a window size equal to the median number. The *r*^2^ threshold was set at 0.8 for the second round of pruning, after which 76,602 SNPs remained that were also found in HapMap. After exclusion of outlier subjects (34 GWAS phase and 37 replication phase samples >3 s.d. values from the median of the first two principal components), we used the first two eigenvectors from the results in these analyses as covariates in the association tests to adjust for any possible population structure among the study subjects (Supplementary Fig. 10). The genomic inflation parameter *λ*_GC1000_ (calculated following de Bakker, *et al*.^62^ was sufficiently small (<1.05 in each of the GWAS performed with the different phenotypes) to suggest that population stratification was adequately controlled in our model and that there was no inflation of false positives from this potential confounder.

### Imputation

We performed genotype imputation using IMPUTE2^63^ (https://mathgen.stats.ox.ac.uk/impute/impute_v2.html) and the 1000 Genomes reference panel^64^. We used a ‘cosmopolitan' 1000 Genomes reference panel for imputation consisting of an intersection of the AFR, EUR, and ASN panels. We first used the strand-checking utility of SHAPEIT v2 (ref. 65) (https://mathgen.stats.ox.ac.uk/genetics_software/shapeit/shapeit.html) to ensure consistent strand assignments between the reference data set and the QC cleaned and filtered data sets, and we subsequently corrected strand and/or removed SNPs where strandedness could not be resolved. We used a conservative allelic *r*^2^ threshold of 0.9 to remove questionable imputed SNPs.

### Association tests

The genotype call probabilities from the filtered IMPUTE2 output were used in a logistic regression model between PCOS case–control status and the genotypes probabilities under an additive model adjusting for BMI, and the first three principal components of ancestry. Similarly, we used a linear regression model between each of the quantitative reproductive hormone levels (treated as continuous variables) and the genotype probabilities under an additive model adjusting for BMI, the first three principal components of ancestry, assay site and assay method. FSH and SHBG were log-transformed to normalize before conducting the association tests. We used the frequentist approach in SNPTEST^66^ v2.2.0 (https://mathgen.stats.ox.ac.uk/genetics_software/snptest/snptest.html) or ProbABEL^67^ v0.4.3 to estimate the odds ratio and 95% confidence intervals (PCOS case/control status), or beta and s.e. values (hormone levels) for each regression model and assess the significance of the association between the SNP and the phenotype of interest.

### Stage 2 Metabochip replication

We attempted replication our top associations in a second set of 1,799 PCOS cases and 1,231 phenotyped controls by adding 12,921 SNPs (those with *P*<1 × 10^−5^ in the GWAS phase, as well as candidate SNPs for ancillary studies) as custom content to the Metabochip^68^. Genotyping was performed at the Broad Institute Centre for Genotyping and Analysis (CGA), Cambridge, MA, who released 9,893 of the 12,921 add-on SNPs attempted. Genotyping QC, assessing ancestry, imputation to 1,000 genomes, and association tests followed that of the GWAS phase described above. To assess population substructure in the Stage 2 Replication, we analysed all unduplicated PCOS replication study samples separately, along with HapMap (Phase 3 CEU, CHB, JPT and YRI) to detect of population outliers among the study. We started with a set of 197,415 autosomal SNPs with missing call rate <2%. From this pool of overlapping SNPs, we selected a subset through two rounds of LD pruning using the pair-wise genotypic correlation method in PLINK. In the first round we remove SNPs in short-range LD using a window size of 50 SNPs with a five SNP offset and an *r*^2^ threshold of 0.8. The second round of pruning removed SNPs in long-range LD using a window size equal to the median number. The *r*^2^ threshold was set at 0.8 for the second round of pruning after which 53,828 SNPs remained that were also found in HapMap. After exclusion of outlier subjects (>2 s.d. values from the median of the first two principal components) we used the first two eigenvectors from the results in these analyses as covariates in the association tests to adjust for any possible population structure among the study subjects (Supplementary Fig. 11).

### Stage 3 (UK) Replication

The Illumina Core Exome and Illumina Human 1.2 Million Duo Custom v1_A platforms were used to genotype the UK cases and controls, respectively. Overlapping variants (*N*=191,563) were included in the quality control (QC) and imputation phases. As cases and controls were typed separately, stringent QC thresholds were applied before and after merging the case–control data. Pre-imputation QC included exclusion filters: (i) HWE *P* value <0.0001 in cases and controls separately, and HWE *P* value<0.01 in the merged data; (ii) SNP call rate<95% in cases and controls separately; (iii) sample call rate <95% in cases and controls separately; (iv) differential SNP missingness between cases and controls in the merged data, *P* value<0.01; (v) s.e.>10 in pre-imputation logistic regression analysis; (vi) MAF<1% in the final clean merged data. In addition, related individuals and ethnic outliers were detected and excluded using PLINK pairwise IBD estimation and multidimensional scaling analysis (MDS).

Pre-phasing and imputation were performed using SHAPEIT and IMPUTE2 with the 1000 Genomes March 2012 (v3) reference panel, respectively. SNPTEST was used for association analyses. Case–control and quantitative analyses included C1 from the PLINK MDS analysis as a covariate to adjust for population stratification. Variants with info score<0.4, s.e.<0 and s.e.>10, and *P*-value<0 were excluded from the association results.

### Meta-analysis

The betas and s.e. values were combined across the GWAS and replication cohorts using meta-analysis under a fixed effects model weighting each strata by sample size. METAL^69^ (http://www.sph.umich.edu/csg/abecasis/metal/index.html) calculates a z-statistic, which summarizes the magnitude and direction of effect for the association of a reference allele selected at each marker. After aligning the SNPTEST output from each of the four cohorts to the same reference allele, a weighted sum of the individual cohort results is used to calculate an overall z-statistic and *P* value. The square root of the cohort specific sample size is used as the proportional weight, and these squared weights sum to 1.

### Predicting transcription activity of the GWAS loci

To investigate the transcriptional potential of the primary GWAS loci identified in the dichotomous and quantitative trait analyses, we examined the histone mark patterns and mRNA levels for each locus using ENCODE ChIP-Seq data, RNASeq (http://genome.ucsc.edu) and GNF Expression Atlas 1 Human Data on Affy U95 Chips http://genome.ucsc.edu). A broad tissue distribution was tested for the expression array and the RNAseq. Multiple tissues contribute to the PCOS phenotype. Although there are no ChiP-Seq ENCODE results from any ovarian tissues, PCOS is also associated with substantial metabolic abnormalities^1^. These abnormalities include defects in adipocyte, skeletal muscle and hepatic insulin action as well as pancreatic β-cell dysfunction 1. Our analysis included on metabolic tissues affected in PCOS (adipose nuclei, adult liver, pancreatic islets and skeletal muscle).

We present chromatin Immunoprecipitation sequencing (ChIP-Seq) results from Encyclopaedia of DNA Elements (ENCODE) Consortium Roadmap Epigenomics Consortium [(http://www.roadmapepigenomics.org/data) for six histone marks (H3K27m3, H3K36m3, H3K4m1, H3K4m3, H3K9ac and H3K9m3) in four human tissues that are potentially relevant to PCOS (adipose nuclei, adult liver, pancreatic islets and skeletal muscle)]. H3K27m3 histone mark is the tri-methylation of lysine 27 of the H3 histone protein and is associated with facultatively expressed genes (www.epigentek.com). H3K36m3, tri-methylation of lysine 36 of H3, is associated with actively transcribed genes (www.epigentek.com). H3K4m1, mono-methylation of lysine 4 of H3, is associated with enhancers and with DNA regions downstream of transcription starts (www.epigentek.com). H3K4m3, tri-methylation of lysine 4 of H3, is associated with promoters that are active or poised to be activated (www.epigentek.com). H3K9ac, acetylation of lysine 9 of H3, is found in actively transcribed promoters (www.epigentek.com). H3K9m3, tri-methylation of lysine 9 of H3, in contrast is found in constitutively repressed genes (www.epigentek.com). We considered a methylation mark to active if there is more than one peak with a height greater than 5 × times background height.

## Additional information

**Accession codes:** Genotype data have been deposited in dbGaP under the accession code phs000368.v1.p1.

**How to cite this article:** Hayes, M. G. *et al*. Genome-wide association of polycystic ovary syndrome implicates alterations in gonadotropin secretion in European ancestry populations. *Nat. Commun.* 6:7502 doi: 10.1038/ncomms8502 (2015).

## Supplementary Material

Supplementary Figures Supplementary Figures 1-11

Supplementary Data 1 Top Associations (P<1E-06 in Phase 1 Discovery GWAS, Stage 2 Replication, or Stage 1+2 Meta-analysis)

Supplementary Data 2 Meta-analysis results after Stage 3 UK Replication of Top 24 Regions after Stage 1+ 2 Meta-Analysis

Supplementary Data 3 c9orf3 PCOS locus adjusted for lead SNPs in the region from Han Chinese PCOS GWAS (Shi *et al*, Nat Genet 44:1020-27, 2012)

Supplementary Data 4 Association results of hormone assay quantitative traits in phenotyped reproductively normal control women for those variants reaching genomewide significant in the PCOS cases.

Supplementary Data 5 Comparison of Stage 1 and Stage 2 results with and without BMI as a covariate.

Supplementary Data 6 eQTLs for the four genomewide significant loci from SCANdb (www.scandb.org)

Supplementary Data 7 Genetic association results of PCOS with and without adjustment for rs11031006

Supplementary Data 8 Replication of known GWAS significant hormone level quantitative traits

Supplementary Data 9 Phenotypic Measures in GWAS samples

Supplementary Data 10 Phenotypic Measures in Replication samples

Supplementary Data 11 Assays Used to Measure Hormone Levels

Supplementary Data 12 Summary of DNA samples and genotyping instances (scans)

Supplementary Data 13 Summary of SNPs attempted, and filtered from the analysis

## Figures and Tables

**Table 1:** Demographic characteristics of PCOS cases and controls in the GWAS and replication.

**Cohort**	**GWAS**						**Replication**					
	**Age (years)**		**BMI (kg m**^−2^)		**Testosterone (ng dl**^−1^)		**Age (years)**		**BMI (kg m**^−2^)		**Testosterone (ng dl**^−1^)	
	**Case**	**Control**	**Case**	**Control**	**Case**	**Control**	**Case**	**Control**	**Case**	**Control**	**Case**	**Controls**
PCOS Family Study*	28 (24–32)*n*=718	—	35.5 (29.1–41.4)*n*=718	—	72 (59–91)*n*=663	—	28 (24–32)*n*=172	29 (24–33)*n*=105	35.0 (28.3–41.5)*n*=172	30.7 (23.3–35.0)*n*=105	70 (59–93)*n*=100	28 (17–35)*n*=35
					63 (44–77)*n*=30	—					68 (49–86)*n*=47	25 (19–32)*n*=70
PPCOSI*	28 (26–30)*n*=64	—	35.3 (27.4–42.1)*n*=64	—	60 (44–82)*n*=62	—	—	—	—	—	—	—
University of Chicago†	29 (25–34)*n*=202	—	36.2 (31.8–42.1)*n*=202	—	68 (52–93)*n*=202	—	27 (23–30)*n*=25	—	38.4 (32.4–42.7)*n*=25	—	64 (50–85)*n*=25	—
NUgene	—	46 (34–58)*n*=2,964	—	25.0 (22.0–29.8)*n*=2,964	—	—	—	—	—	—	—	—
Cedars–Sinai Medical Center‡	—	—	—	—	—	—	26 (22–31)*n*=130	37 (27–42)*n*=17	27.7 (22.5–33.9)*n*=130	24.6 (21.5–31.5)*n*=17	43 (31–62)*n*=121	23 (17–29)*n*=11
Massachusetts General Hospital	—	—	—	—	—	—	28 (24–32)*n*=472	25 (22–31)*n*=402	29.0 (23.9–37.0)*n*=472	23.0 (21.1–25.0)*n*=402	—	—
Medical College of Wisconsin*	—	—	—	—	—	—	—	38 (32–41)*n*=452	—	31.9 (24.2–38.9)*n*=452	—	19 (15–24)*n*=452
PPCOSII*	—	—	—	—	—	—	28 (26–31)*n*=265	—	35.1 (26.6–41.8)*n*=265	—	50 (37–67)*n*=265	—
University of Alabama at Birmingham§	—	—	—	—	—	—	27 (22–33)*n*=193	32 (26–36)*n*=91	34.1 (27.8–40.8)*n*=193	23.7 (21.7–27.7)*n*=91	77 (63–94)*n*=193	44 (32–56)*n*=81
University of Athens||	—	—	—	—	—	—	23 (20–28)*n*=542	26 (23–34)*n*=164	24.5 (21.4–30.9)*n*=542	22.0 (20.1–24.5)*n*=164	72 (61–89)*n*=542	31 (25–43)*n*=163
Imperial College London/University of Oxford¶	—	—	—	—	—	—	32 (28–36)*n*=222	45 (45–45)*n*=1,335	28.4 (23.2–36.5)*n*=217	25.5 (23.0–29.4)*n*=1,335	69 (53–86)*n*=223	—

PCOS, polycystic ovary syndrome; BMI, body mass index; GWAS, genome-wide association studies. Data are expressed as median (25th–75th interquartile range). Hormone results are separated by assay method when multiple methods and labs were used within a cohort, however the normal range was identical.

^*^Testosterone normal range<59 ng dl^**−**1^

^†^Testosterone normal range<71 ng dl^**−**1^.

^‡^Testosterone normal range<35 ng dl^**−**1^.

^§^Testosterone normal range<85 ng dl^**−**1^.

^||^Testosterone normal range<60 ng dl^**−**1^.

^¶^Testosterone normal range<70 ng dl^**−**1^

**Table 2:**
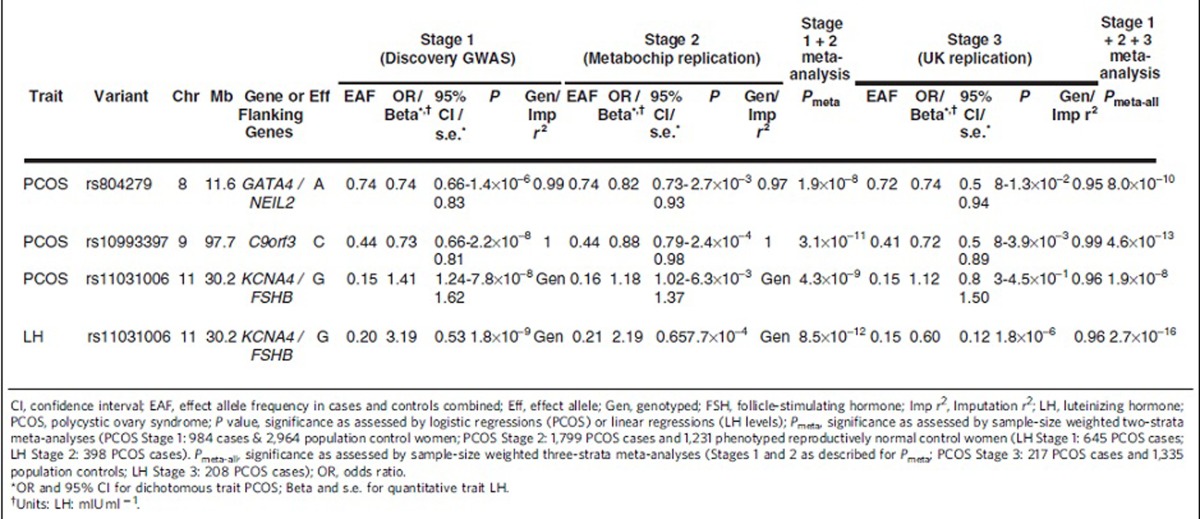
Genome-wide significant associations of PCOS and associated reproductive hormonal quantitative traits.

CI, confidence interval; EAF, effect allele frequency in cases and controls combined; Eff, effect allele; Gen, genotyped; FSH, follicle-stimulating hormone; Imp *r*^2^, Imputation *r*^2^; LH, luteinizing hormone; PCOS, polycystic ovary syndrome; *P* value, significance as assessed by logistic regressions (PCOS) or linear regressions (LH levels); *P*_meta_, significance as assessed by sample-size weighted two-strata meta-analyses (PCOS Stage 1: 984 cases & 2,964 population control women; PCOS Stage 2: 1,799 PCOS cases and 1,231 phenotyped reproductively normal control women (LH Stage 1: 645 PCOS cases; LH Stage 2: 398 PCOS cases). *P*_meta-all_, significance as assessed by sample-size weighted three-strata meta-analyses (Stages 1 and 2 as described for *P*_meta_; PCOS Stage 3: 217 PCOS cases and 1,335 population controls; LH Stage 3: 208 PCOS cases); OR, odds ratio.

^*^OR and 95% CI for dichotomous trait PCOS; Beta and s.e. for quantitative trait LH.

^†^Units: LH: mIU ml^−1^.

**Table 3:**
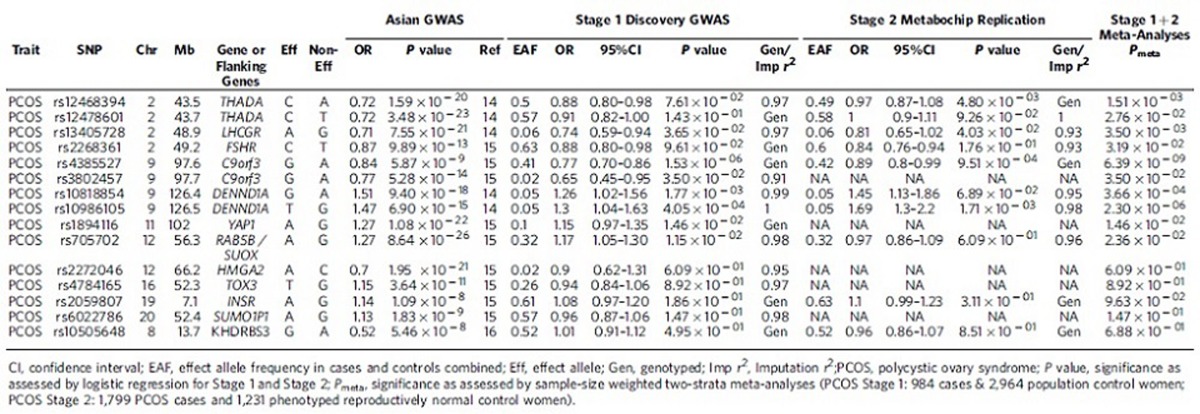
Genetic association results of variants identified in previous PCOS GWAS.
