# Corrigendum: Genome-wide association of polycystic ovary syndrome implicates alterations in gonadotropin secretion in European ancestry populations

**DOI:** 10.1038/ncomms10762

**Published:** 2016-02-12

**Authors:** M. Geoffrey Hayes, Margrit Urbanek, David A. Ehrmann, Loren L. Armstrong, Ji Young Lee, Ryan Sisk, Tugce Karaderi, Thomas M. Barber, Mark I. McCarthy, Stephen Franks, Cecilia M. Lindgren, Corrine K. Welt, Evanthia Diamanti-Kandarakis, Dimitrios Panidis, Mark O. Goodarzi, Ricardo Azziz, Yi Zhang, Roland G. James, Michael Olivier, Ahmed H. Kissebah, Reproductive Medicine Network, Ruben Alvero, Huiman X. Barnhart, Valerie Baker, Kurt T. Barnhart, G. Wright Bates, Robert G. Brzyski, Bruce R. Carr, Sandra A. Carson, Peter Casson, Nicholas A. Cataldo, Gregory Christman, Christos Coutifaris, Michael P. Diamond, Esther Eisenberg, Gabriella G. Gosman, Linda C. Giudice, Daniel J. Haisenleder, Hao Huang, Stephen A. Krawetz, Scott Lucidi, Peter G. McGovern, Evan R. Myers, John E. Nestler, Dana Ohl, Nanette Santoro, William D. Schlaff, Peter Snyder, Michael P. Steinkampf, J. C. Trussell, Rebecca Usadi, Qingshang Yan, Heping Zhang, Elisabet Stener-Victorin, Richard S. Legro, Andrea Dunaif

Nature Communications 6: Article number: 7502 10.1038/ncomms8502 (2015); Published: 08182015; Updated: 02122016

In the original version of this Article the genetic locus 8p23.1 was incorrectly referred to as 8p32.1 throughout. For example, in the Abstract, the sentence beginning ‘Three loci reach genome-wide significance in the case-control meta-analysis…' originally read ‘Three loci reach genome-wide significance in the case-control meta-analysis, two novel loci mapping to chr 8p32.1 and chr 11p14.1, and a chr 9q22.32 locus previously found in Chinese PCOS'. Each occurrence of this error has now been corrected in both the PDF and HTML versions of the Article.

