# Publisher Correction: Genome-wide association of polycystic ovary syndrome implicates alterations in gonadotropin secretion in European ancestry populations

**DOI:** 10.1038/s41467-020-15793-w

**Published:** 2020-04-28

**Authors:** M. Geoffrey Hayes, Margrit Urbanek, David A. Ehrmann, Loren L. Armstrong, Ji Young Lee, Ryan Sisk, Tugce Karaderi, Thomas M. Barber, Mark I. McCarthy, Stephen Franks, Cecilia M. Lindgren, Corrine K. Welt, Evanthia Diamanti-Kandarakis, Dimitrios Panidis, Mark O. Goodarzi, Ricardo Azziz, Yi Zhang, Roland G. James, Michael Olivier, Ahmed H. Kissebah, Reproductive Medicine Network, Ruben Alvero, Huiman X. Barnhart, Valerie Baker, Kurt T. Barnhart, G. Wright Bates, Robert G. Brzyski, Bruce R. Carr, Sandra A. Carson, Peter Casson, Nicholas A. Cataldo, Gregory Christman, Christos Coutifaris, Michael P. Diamond, Esther Eisenberg, Gabriella G. Gosman, Linda C. Giudice, Daniel J. Haisenleder, Hao Huang, Stephen A. Krawetz, Scott Lucidi, Peter G. McGovern, Evan R. Myers, John E. Nestler, Dana Ohl, Nanette Santoro, William D. Schlaff, Peter Snyder, Michael P. Steinkampf, J. C. Trussell, Rebecca Usadi, Qingshang Yan, Heping Zhang, Elisabet Stener-Victorin, Richard S. Legro, Andrea Dunaif

**Affiliations:** 1 Division of Endocrinology, Metabolism, and Molecular Medicine, Department of Medicine, Northwestern University Feinberg School of Medicine, Chicago, Illinois 60611 USA; 2 Center for Genetic Medicine, Northwestern University Feinberg School of Medicine, Chicago, Illinois 60611 USA; 3 Department of Anthropology, Northwestern University, Evanston, Illinois 60208 USA; 4 Section of Endocrinology, Diabetes, and Metabolism, The University of Chicago, Chicago, Illinois 60637 USA; 5 Wellcome Trust Centre for Human Genetics, University of Oxford, Oxford, OX3 7BN UK; 6 Warwick Medical School, University of Warwick, Warwick, CV4 7AL UK; 7 Oxford Centre for Diabetes, Endocrinology and Metabolism, University of Oxford, Oxford, OX3 7LE UK; 8 Oxford NIHR Biomedical Research Centre, Churchill Hospital, Headington, OX3 7LE UK; 9 Institute of Reproductive & Developmental Biology, Hammersmith Hospital, Imperial College London, London, W12 0NN UK; 10 Program in Medical and Population Genetics, Broad Institute of Harvard and MIT, Cambridge, Massachusetts 02142 USA; 11 Division of Endocrinology, Metabolism and Diabetes, University of Utah, Salt Lake City, Utah 84112 USA; 12 Endocrinology and Metabolism, University of Athens Medical School, Athens, 115 27 Greece; 13 Division of Endocrinology and Human Reproduction, 2nd Department of Obstetrics and Gynecology, Aristotle University of Thessaloniki, Thessaloniki, 54124 Greece; 14 Division of Endocrinology, Diabetes and Metabolism, Department of Medicine, Cedars-Sinai Medical Center, Los Angeles, California 90048 USA; 15 Departments of Obstetrics and Gynecology and Medicine, Medical College of Georgia, Georgia Regents University, Augusta, Georgia 30912 USA; 16 TOPS Obesity and Metabolic Research Center, Department of Medicine, Medical College of Wisconsin, Milwaukee, Wisconsin 53226 USA; 17 Human and Molecular Genetics Center, Medical College of Wisconsin, Milwaukee, Wisconsin 53226 USA; 18 Department of Genetics, Texas Biomedical Research Institute, San Antonio, Texas 78256 USA; 19 Department of Physiology and Pharmacology, Karolinska Institutet, SE-171 77 Stockholm, Sweden; 20 Department of Obstetrics and Gynecology, Penn State College of Medicine, Hershey, Pennsylvania 17033 USA; 21 Department of Obstetrics and Gynecology, University of Colorado, Aurora, Colorado 80045 USA; 22 Department of Biostatistics & Bioinformatics, Duke University Medical Centre, Durham, North Carolina 27710 USA; 23 Department of Obstetrics and Gynecology, Stanford University Medical Centre, Stanford, California 94304 USA; 24 Department of Obstetrics and Gynecology, University of Pennsylvania School of Medicine, Philadelphia, Pennsylvania 19104 USA; 25 Department of Obstetrics and Gynecology, University of Alabama, Birmingham, Alabama 35249 USA; 26 Department of Obstetrics and Gynecology, University of Texas Health Science Centre, San Antonio, Texas 78229 USA; 27 Department of Obstetrics and Gynecology, University of Texas Southwestern Medical Center, Dallas, Texas 75390 USA; 28 Department of Obstetrics and Gynecology, Baylor College of Medicine, Houston, Texas 77030 USA; 29 Department of Obstetrics and Gynecology, University of Vermont, Burlington, Vermont, 05495 USA; 30 Department of Obstetrics and Gynecology, University of Michigan, Ann Arbor, Michigan 48109 USA; 31 Department of Obstetrics and Gynecology, Wayne State University, Detroit, Michigan 48201 USA; 32 Fertility and Infertility Branch, Eunice Kennedy Shriver National Institute of Child Health and Human Development, Rockville, Maryland 20852 USA; 33 Department of Obstetrics Gynecology, and Reproductive Sciences, University of Pittsburgh, Pittsburgh, Pennsylvania 15213 USA; 34 Department of Obstetrics Gynecology, and Reproductive Sciences, University of California at San Francisco, San Francisco, California 94143 USA; 35 Ligand Core Laboratory, University of Virginia Center for Research in Reproduction, Charlottesville, Virginia 22908 USA; 36 Department of Biostatistics, Yale University School of Public Health, New Haven, Connecticut 06520 USA; 37 Department of Obstetrics and Gynecology, Virginia Commonwealth University, Richmond, Virginia 23298 USA; 38 Department of Obstetrics, Gynecology and Women’s Health, University of Medicine and Dentistry of New Jersey, Newark, New Jersey 07103 USA; 39 Department of Obstetrics and Gynecology, Duke University Medical Center, Durham, North Carolina 27710 USA; 40 Department of Internal Medicine, Virginia Commonwealth University School of Medicine, Richmond, Virginia 23298 USA; 41 Department of Urology, State University of New York Upstate Medical University, Onondaga, New York 13210 USA; 42 Carolinas Medical Center, Charlotte, North Carolina 28204 USA

Correction to: *Nature Communications* 10.1038/ncomms8502, published online 18 August 2015.

The original version of the Supplementary Data associated with this Article duplicated Supplementary Data 10 for both Supplementary Data 9 and Supplementary Data 10. The correct version of Supplementary Data 9 is provided below.

## Supplementary information

Supplementary Data 9

