# Supplementary material for: Genome-wide association of polycystic ovary syndrome implicates alterations in gonadotropin secretion in European ancestry populations
Source: Nat Commun. 2015 Aug 18;6:7502. doi: 10.1038/ncomms8502 (PMC4557132; doi:10.1038/ncomms8502)
Supplement: Supplementary Figures — 1-11 [file ncomms8502-s1.pdf]

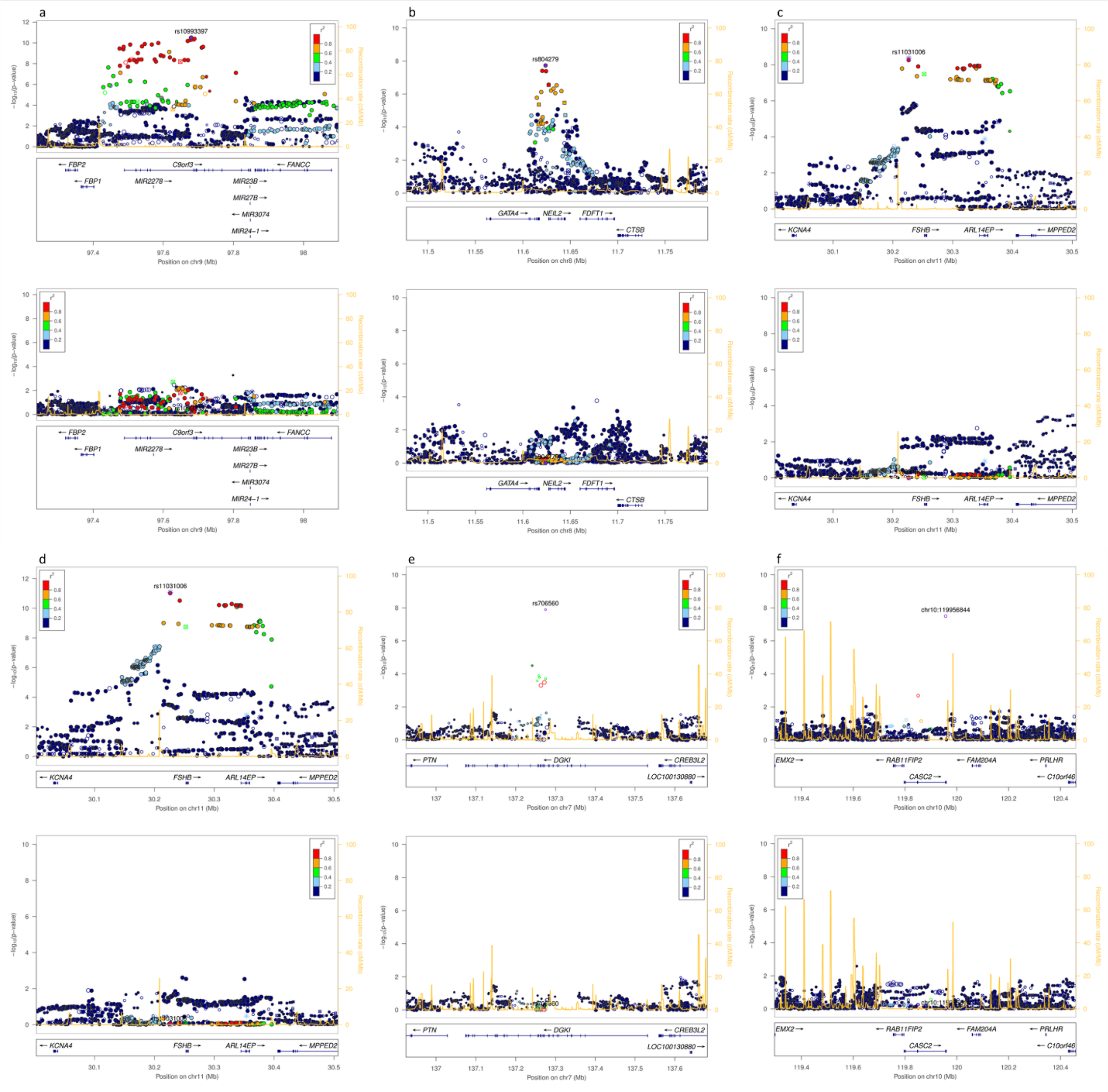

Supplementary Figure 1: Attenuation of association signals after conditioning for the lead SNP. a) attenuation of association signal at the 9p22.32 PCOS locus after conditioning for the lead SNP rs10993397; b) attenuation of association signal at the 8p32.1 PCOS locus after conditioning for the lead SNP rs804279; c) attenuation of association signal at the 11p14.1 PCOS locus after conditioning for the lead SNP rs11031006; d) attenuation of association signal at the 11p14.1 LH locus after conditioning for the lead SNP rs11031006; e) attenuation of association signal at the 7q33 Testosterone locus after conditioning for the lead SNP rs706560; f) attenuation of association signal at the 10q26.11 FSH locus after conditioning for the lead variant chr10:119956844:I. In each panel, the top locuszoom plots (see Figure 2 for description) is without conditioning on lead SNP and the bottom locuszoom plot is after conditioning on lead SNP (in purple). For (a-c) PCOS loci, P-values are from sample-size weighted two-strata meta-analysis of strata-specific logistic regression P-values (Stage 1: 984 cases & 2,964 population control women; Stage 2: 1,799 PCOS cases and 1,231 phenotyped

reproductively normal control women). For (d-f) P-values are from sample-size weighted two-strata meta-analysis of strata-specific linear regression P-values (d: LH level locus, Stage 1: 645 PCOS cases, Stage 2: 399 PCOS cases; e: T level locus, Stage 1: 957 PCOS cases; Stage 2: 1,293 PCOS cases; f) FSH level locus, Stage 1: 647 PCOS cases; Stage 2: 398 PCOS cases).

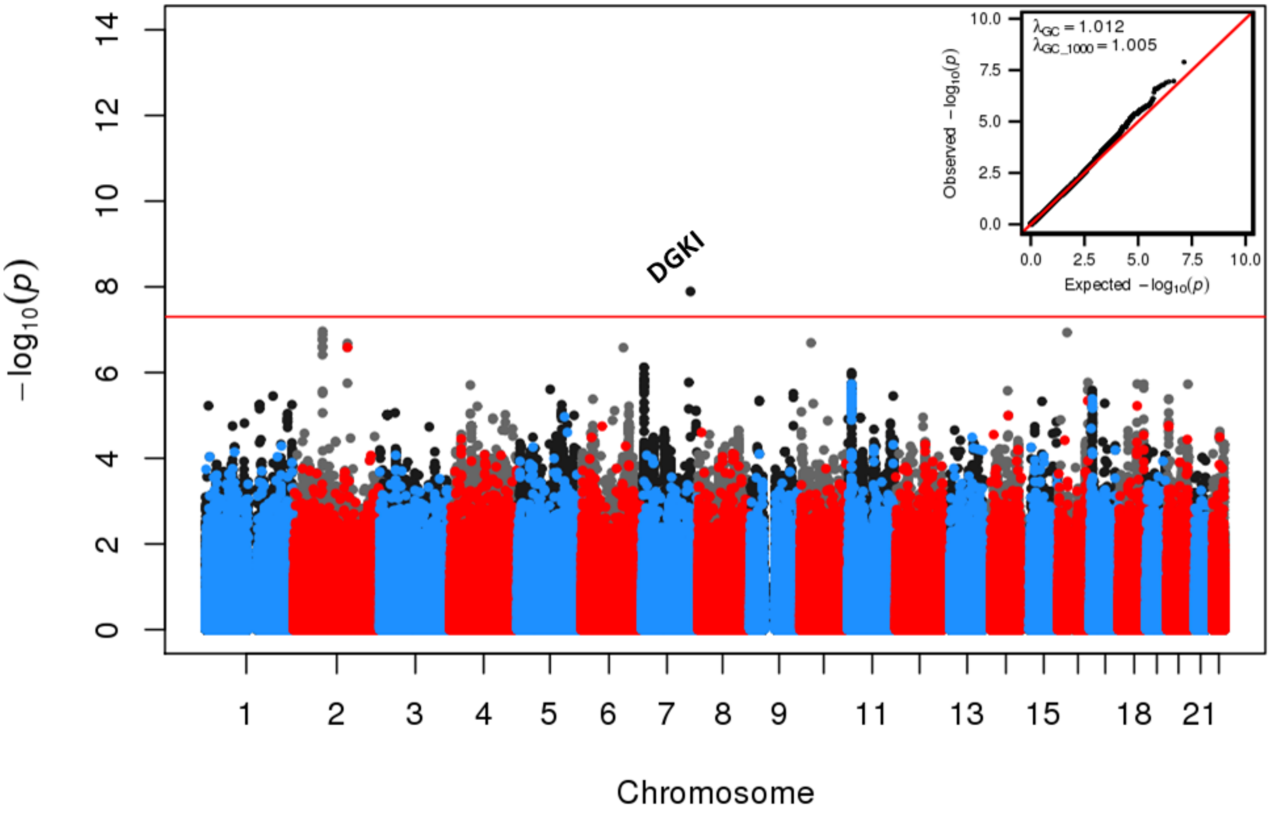

Supplementary Figure 2: Manhattan and QQ plots for total T. Alternating blue and red colors indicate genotyped SNPs, and accompanying black and grey colors indicate imputed variants, on odd and even chromosomes respectively. The red horizontal red line indicates genomewide significance. QQ plots and λ_{GC} are inset in the upper right corner of each plot. P-values are from sample-size weighted two-strata meta-analysis of strata-specific linear regression P-values (Stage 1: 957 PCOS cases; Stage 2: 1,293 PCOS cases).

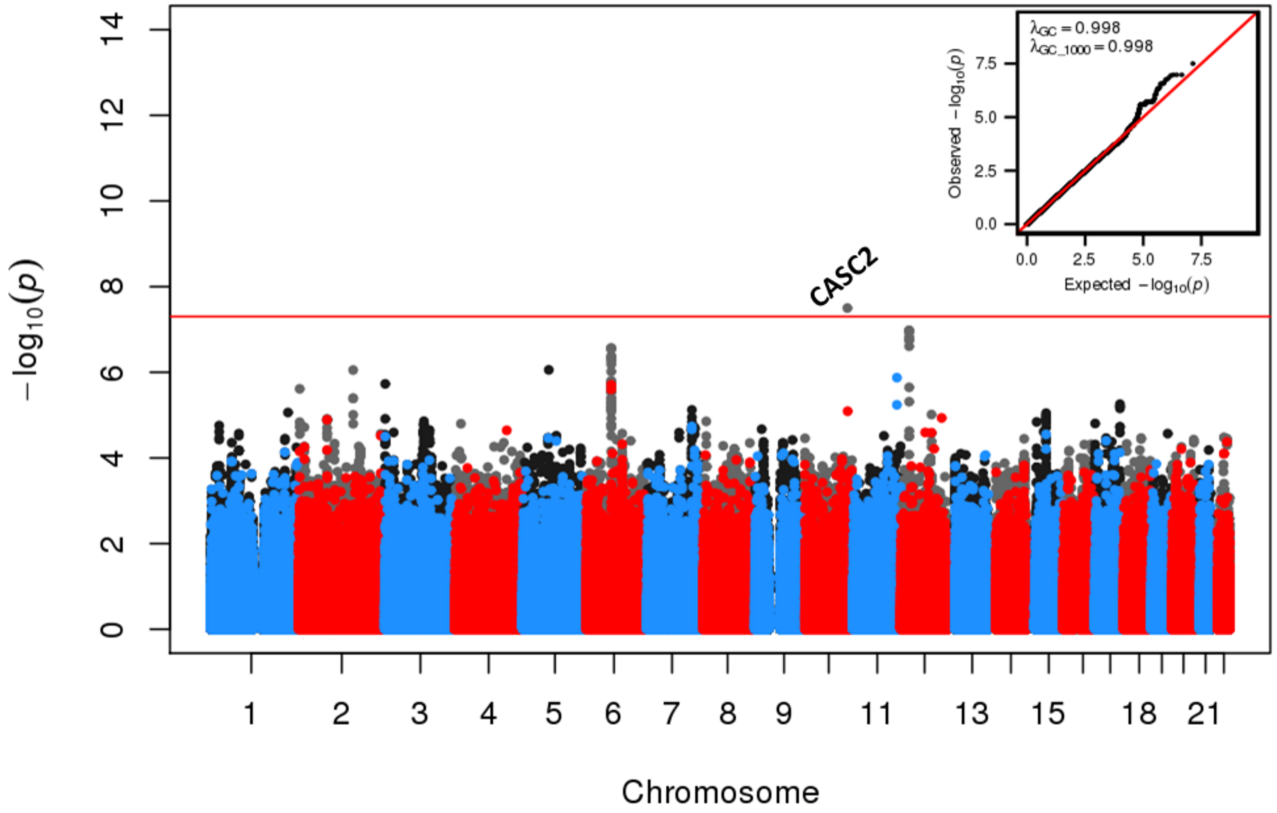

Supplementary Figure 3: Manhattan and QQ plots for $\log_{10}$(FSH). Alternating blue and red colors indicate genotyped SNPs, and accompanying black and grey colors indicate imputed variants, on odd and even chromosomes respectively. The red horizontal red line indicates genomewide significance. QQ plots and λ_{GC} are inset in the upper right corner of each plot. P-values are from sample-size weighted two-strata meta-analysis of strata-specific linear regression P-values (Stage 1: 647 PCOS cases; Stage 2: 398 PCOS cases).

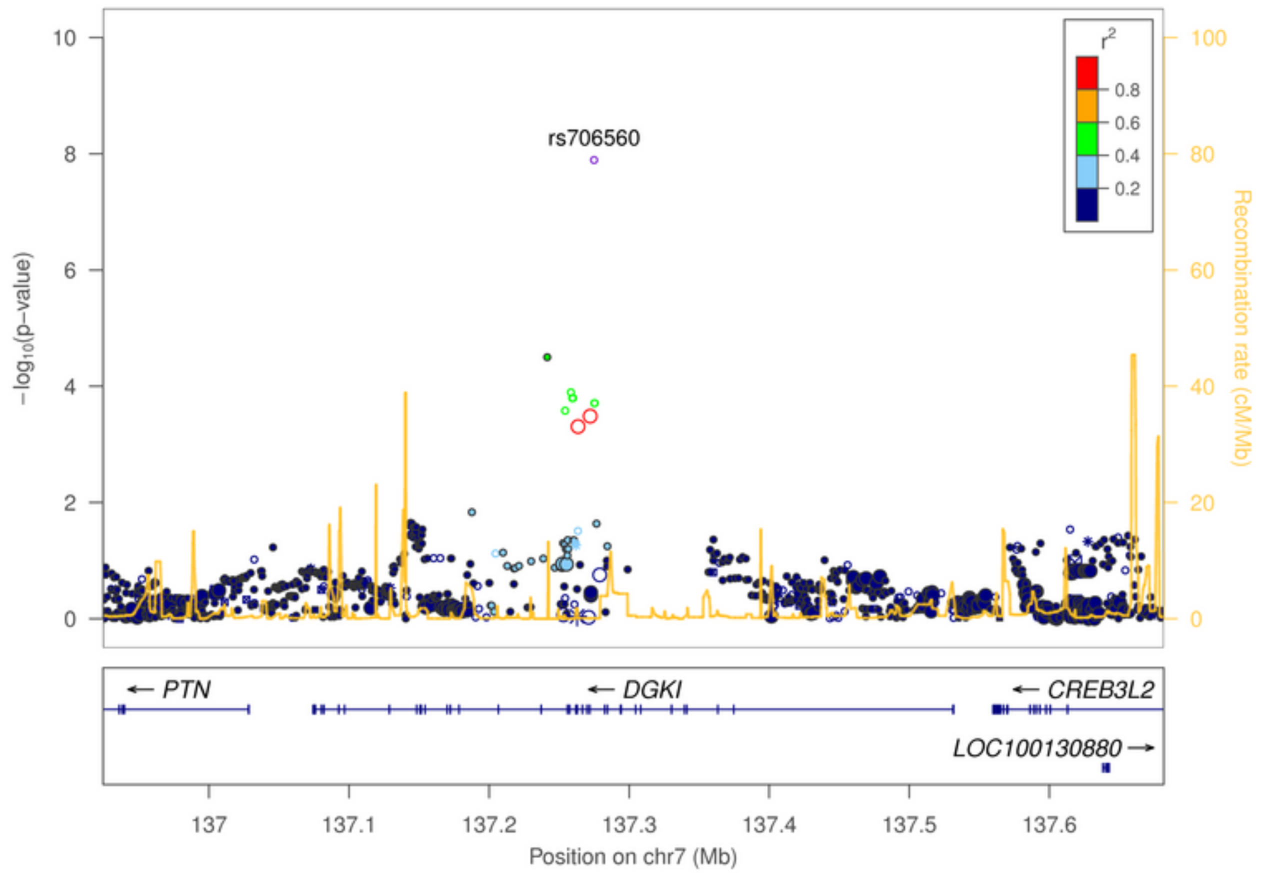

Supplementary Figure 4. Locuszoom plot of association results, linkage disequilibrium and recombination rates around the top GWAS association results for Chr. 7 T locus (*DGKI*). The top panel reflects the meta-analysis results of the combined GWAS and replication phases. The LD estimates are color coded as a heatmap from dark blue ($0 \geq r^2 > 0.2$) to red ($0.8 \geq r^2 > 1.0$). Recombination hotspots are indicated by the yellow lines [recombination rate in cM Mb^{-1} from HapMap (http://hapmap.ncbi.nlm.nih.gov/downloads/recombination/2008-03_rel22_B36/rates/)]. The bottom panel shows the genes and their orientation for each region. P-values are from sample-size weighted two-strata meta-analysis of strata-specific linear regression P-values (Stage 1: 957 PCOS cases; Stage 2: 1,293 PCOS cases).

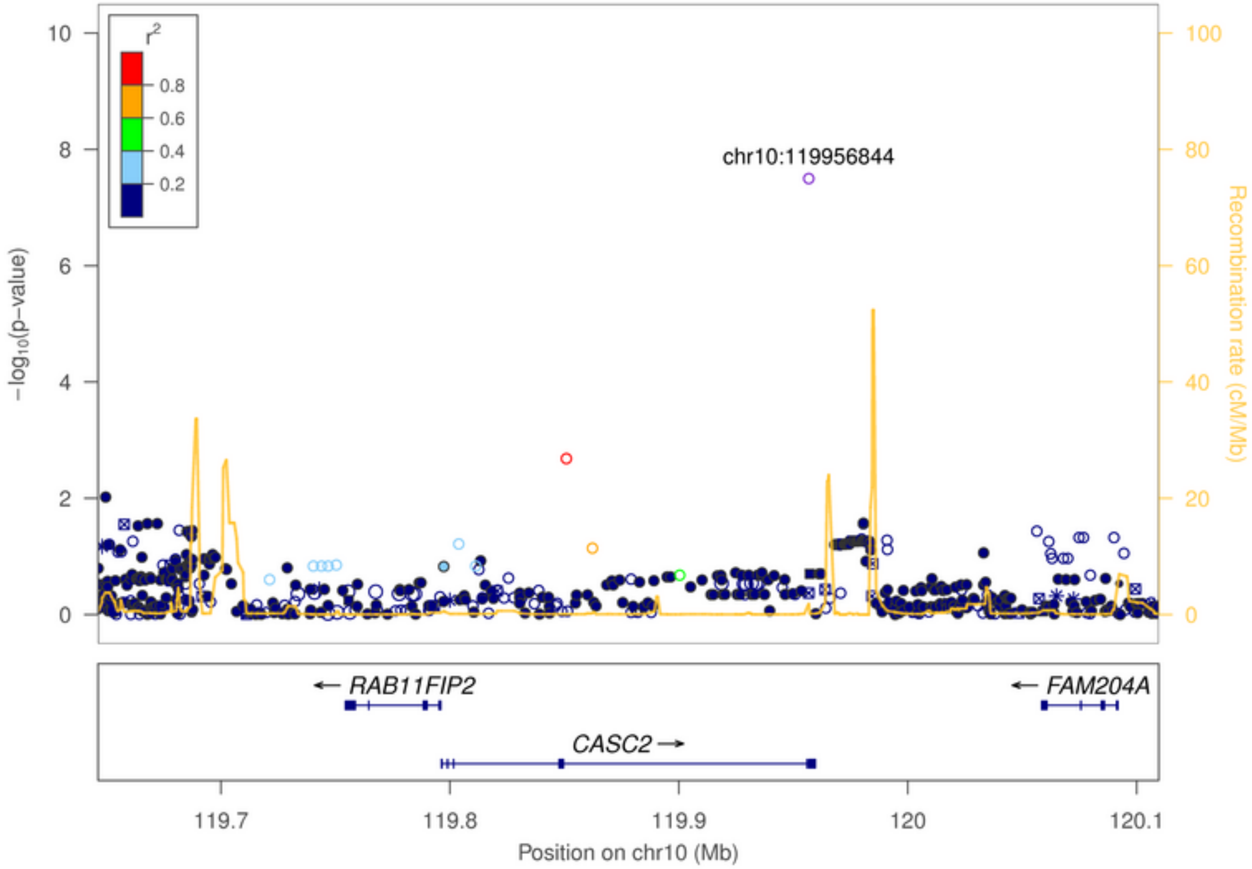

Supplementary Figure 5. Locuszoom plot of association results, linkage disequilibrium and recombination rates around the top GWAS association results for Chr. 10 FSH locus (*CASC2*). The top panel reflects the meta-analysis results of the combined GWAS and replication phases. The LD estimates are color coded as a heatmap from dark blue ($0 \geq r^2 > 0.2$) to red ($0.8 \geq r^2 > 1.0$). Recombination hotspots are indicated by the yellow lines [recombination rate in cM Mb^{-1} from HapMap (http://hapmap.ncbi.nlm.nih.gov/downloads/recombination/2008-03_rel22_B36/rates/)]. The bottom panel shows the genes and their orientation for each region. P-values are from sample-size weighted two-strata meta-analysis of strata-specific linear regression P-values (Stage 1: 647 PCOS cases; Stage 2: 398 PCOS cases).

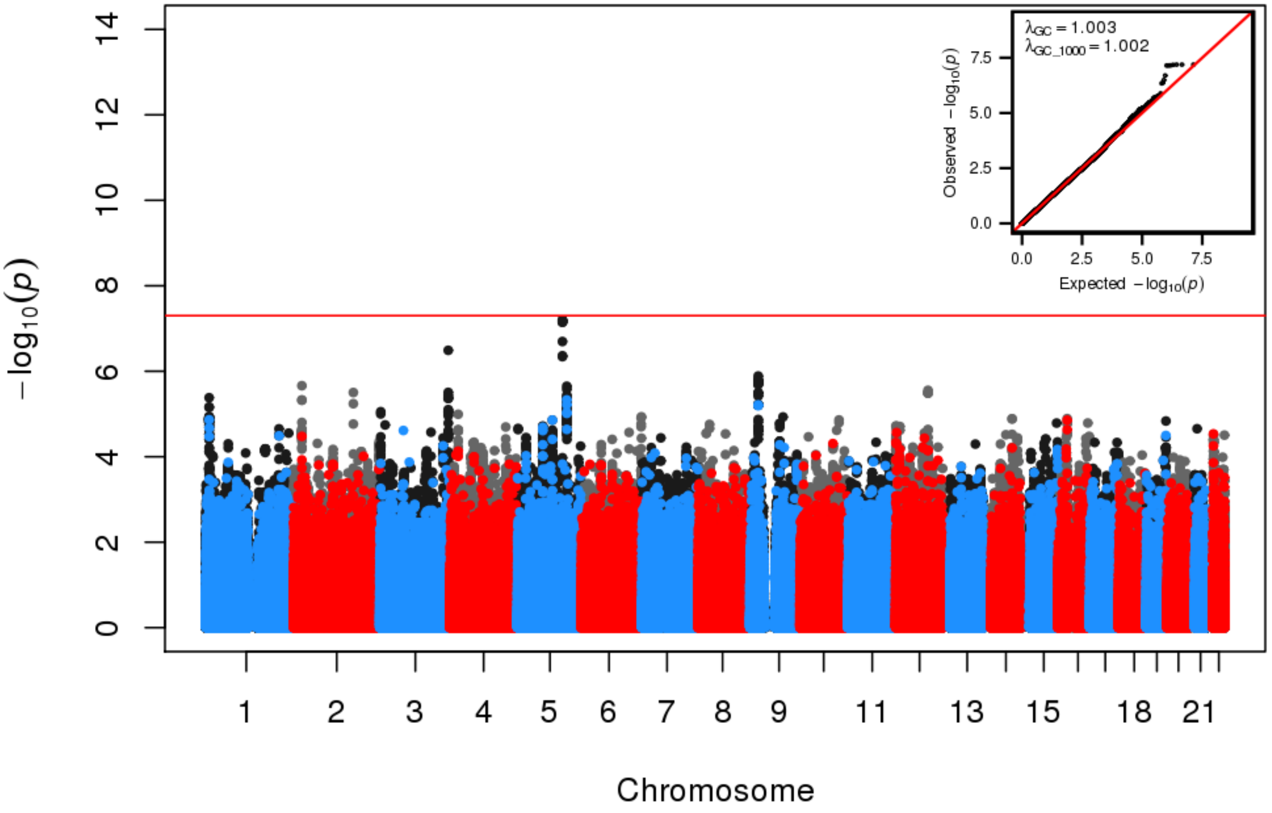

Supplementary Figure 6: Manhattan and QQ plots for DHEAS. Alternating blue and red colors indicate genotyped SNPs, and accompanying black and grey colors indicate imputed variants, on odd and even chromosomes respectively. The red horizontal red line indicates genomewide significance. QQ plots and λ_{GC} are inset in the upper right corner of each plot. P-values are from sample-size weighted two-strata meta-analysis of strata-specific linear regression P-values (Stage 1: 884 PCOS cases; Stage 2: 978 PCOS cases).

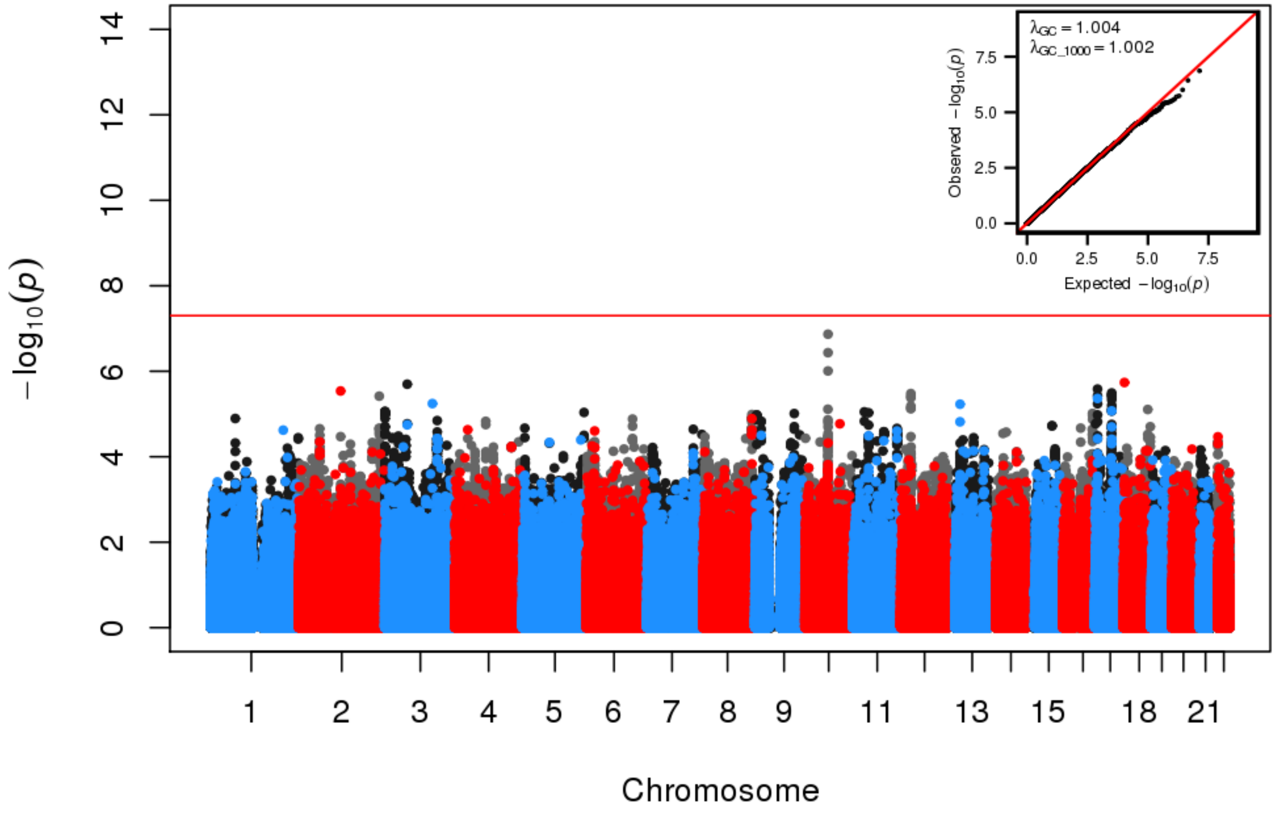

Supplementary Figure 7: Manhattan and QQ plots for $\log_{10}$(SHBG). Alternating blue and red colors indicate genotyped SNPs, and accompanying black and grey colors indicate imputed variants, on odd and even chromosomes respectively. The red horizontal red line indicates genomewide significance. QQ plots and λ_{GC} are inset in the upper right corner of each plot. P-values are from sample-size weighted two-strata meta-analysis of strata-specific linear regression P-values (Stage 1: 903 PCOS cases; Stage 2: 953 PCOS cases).

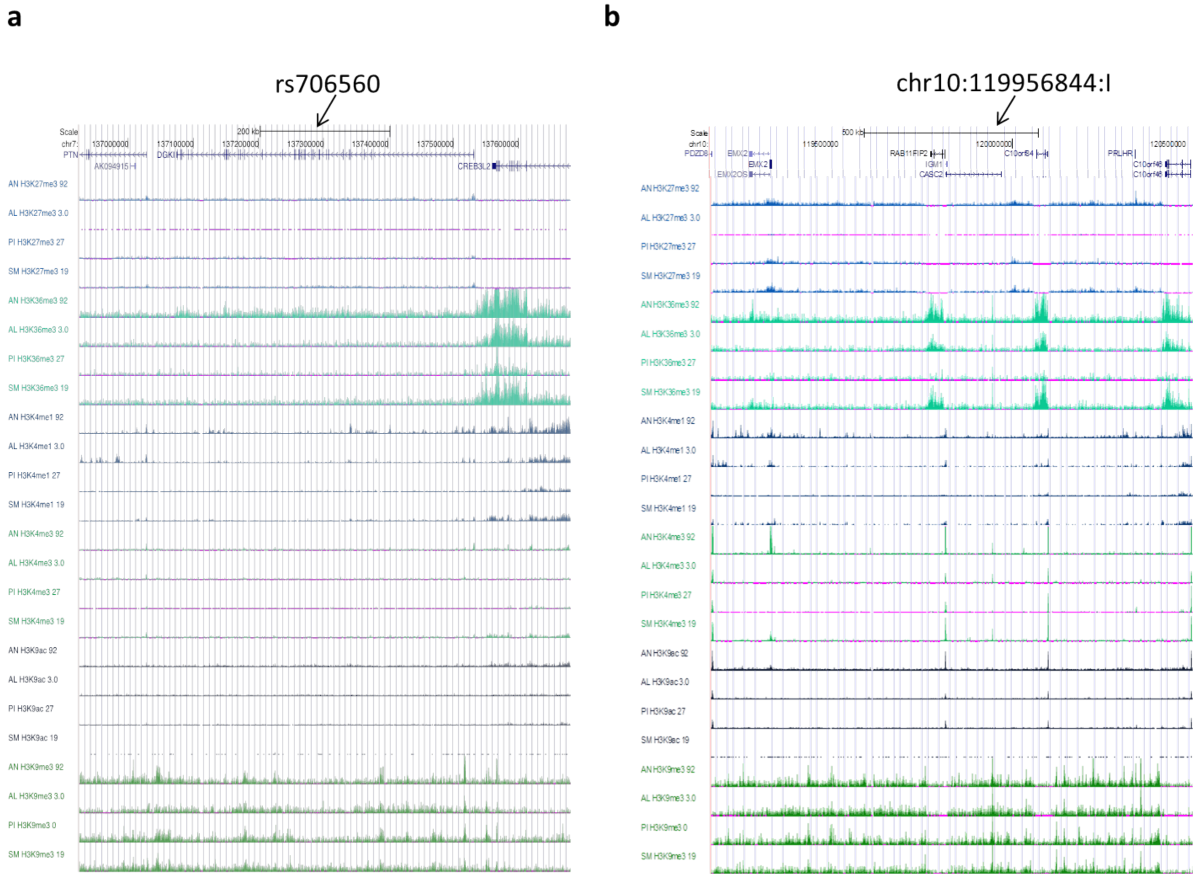

Supplementary Figure 8: ENCODE histone mark profile for GWAS signals genomewide significant after Stages 1 and 2 (http://www.roadmapepigenomics.org/data). Methylation marks in four PCOS relevant human tissue mapping to the a. Chr.7 total T level locus (*DGKI*), and b, Chr. 10 FSH level locus (*CASC2*). The top panel for each genomic region is a schematic of the genomic region including diagrams of genes mapping to the region. ChipSeq signal tracks for each tissue (adipose nuclei =AN, adult liver = AL, pancreatic islets = PI, and skeletal muscle = SM) for six histone marks (H3K27m3, H3K36m3, H3K4m1, H3K4m3, H3K9ac, and H3K9m3). ChipSeq signal tracks are labeled to the left of the panel.

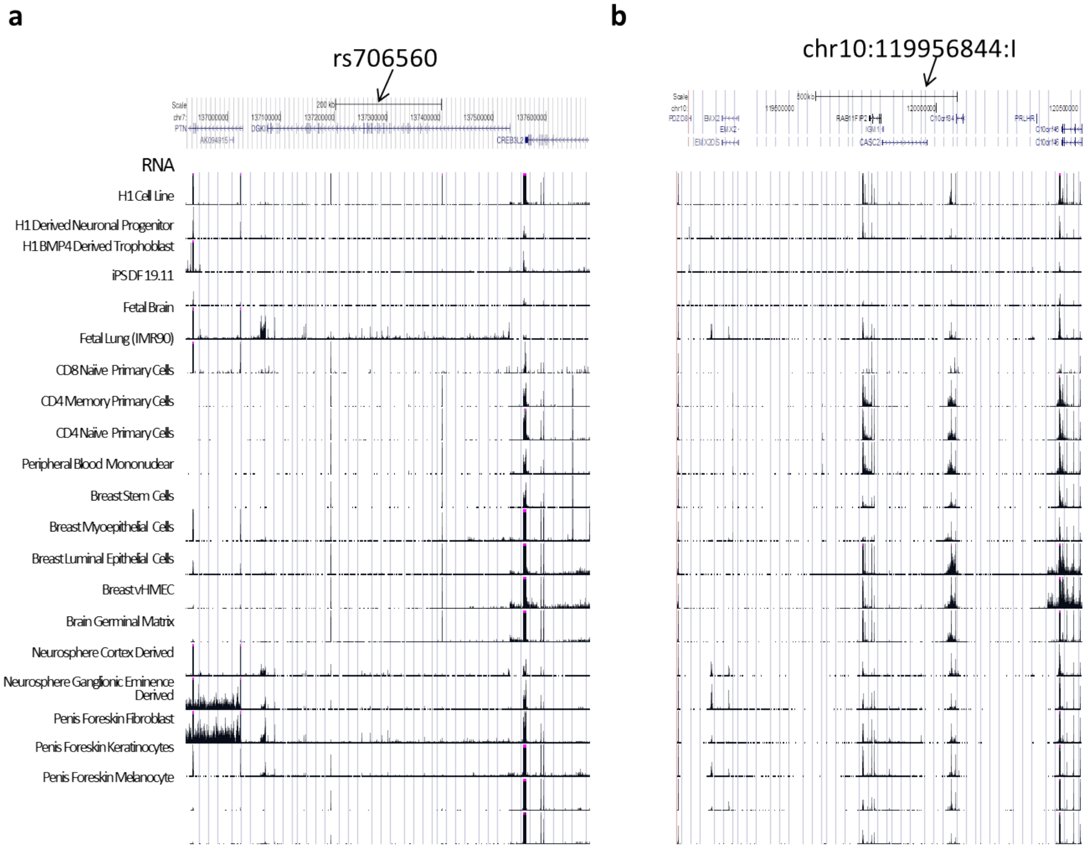

Supplementary Figure 9: ENCODE mRNA levels for GWAS signals genomewide significant after Stages 1 and 2. RNAseq levels in four PCOS relevant human tissue mapping to the a. Chr.7 total T level locus (*DGKI*), and b, Chr. 10 FSH level locus (*CASC2*). The top panel for each genomic region is a schematic of the genomic region including diagrams of genes mapping to the region. Source of RNA are labeled to the left of the panel.

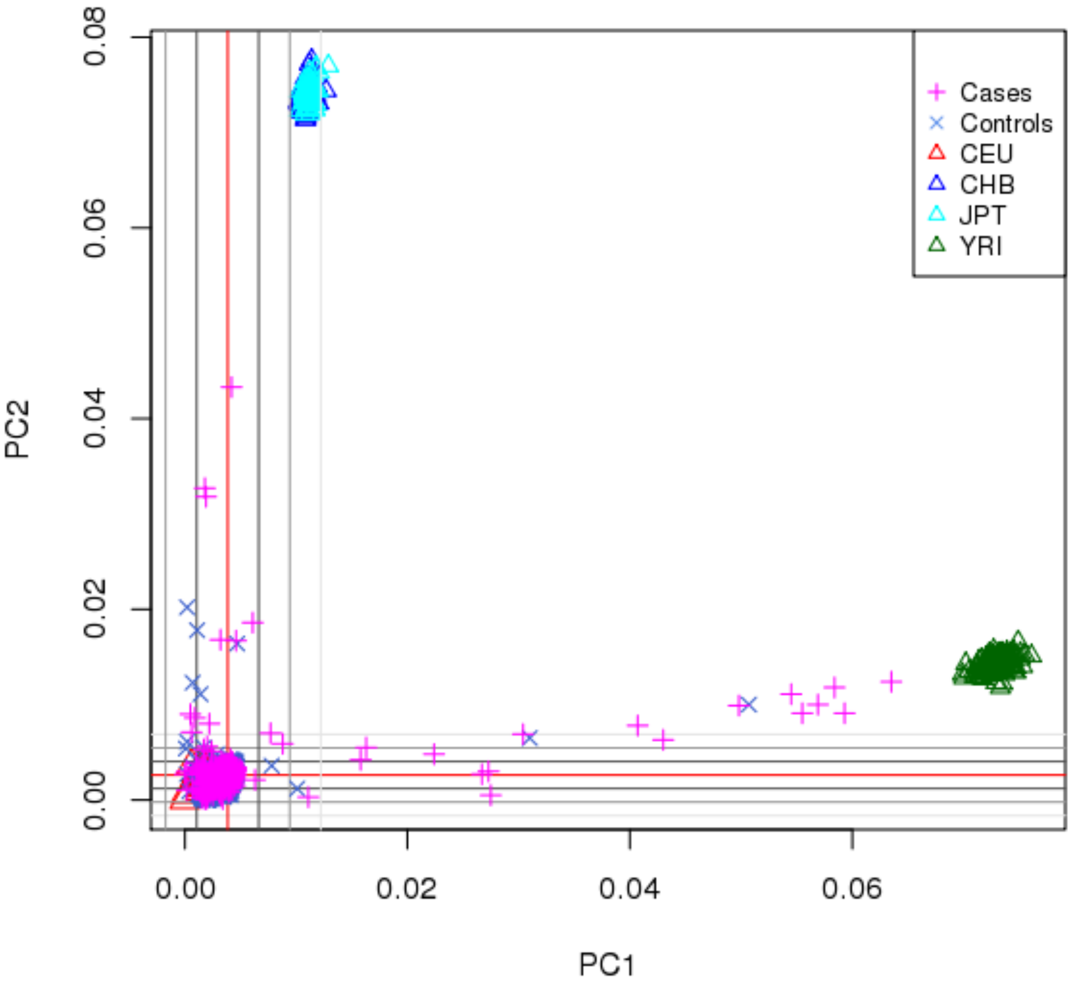

Supplementary Figure 10. Principal components analysis of all unduplicated GWAS study participants (N=984 PCOS cases, 2,964 population controls) with external HapMap controls (N=88).

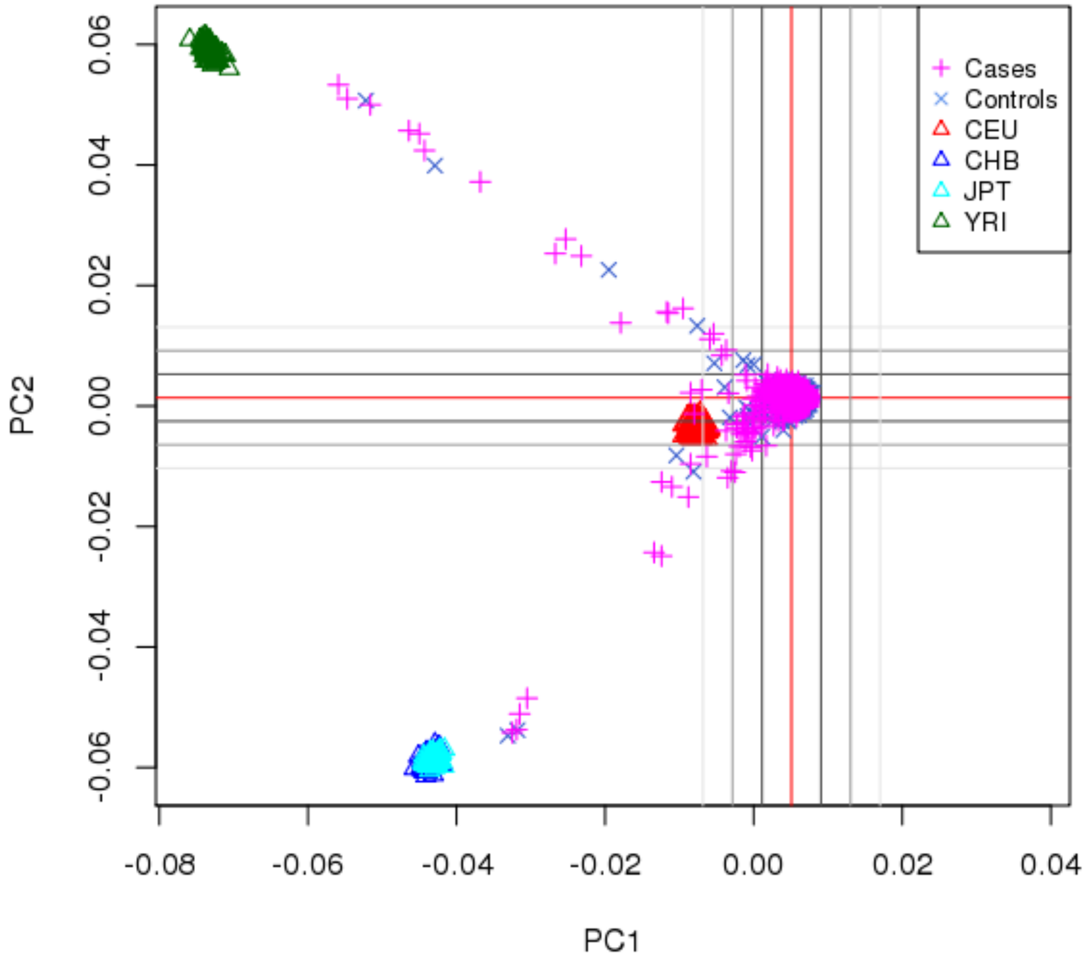

Supplementary Figure 11. Principal components analysis of all unduplicated replication study participants (N= 1,799 PCOS cases and 1,231 phenotyped reproductively normal control women) with external HapMap controls (N=50).
